# Spatio-temporal monitoring of deep-sea communities using metabarcoding of sediment DNA and RNA

**DOI:** 10.7717/peerj.2807

**Published:** 2016-12-21

**Authors:** Magdalena Guardiola, Owen S. Wangensteen, Pierre Taberlet, Eric Coissac, María Jesús Uriz, Xavier Turon

**Affiliations:** 1 Department of Marine Ecology, Centre for Advanced Studies of Blanes (CEAB-CSIC), Blanes, Spain; 2 Department of Animal Biology and Biodiversity Research Institute (IRBIO), University of Barcelona, Barcelona, Spain; 3 Ecosystems & Environment Research Centre, School of Environment & Life Sciences, University of Salford, Salford, United Kingdom; 4 Laboratoire d’Ecologie Alpine (LECA), Centre National de la Recherche Scientifique and Université Grenoble-Alpes, Grenoble, France

**Keywords:** Sediments, eDNA, 18S, eRNA, Meiofauna, Submarine canyons, Biomonitoring

## Abstract

We assessed spatio-temporal patterns of diversity in deep-sea sediment communities using metabarcoding. We chose a recently developed eukaryotic marker based on the v7 region of the 18S rRNA gene. Our study was performed in a submarine canyon and its adjacent slope in the Northwestern Mediterranean Sea, sampled along a depth gradient at two different seasons. We found a total of 5,569 molecular operational taxonomic units (MOTUs), dominated by Metazoa, Alveolata and Rhizaria. Among metazoans, Nematoda, Arthropoda and Annelida were the most diverse. We found a marked heterogeneity at all scales, with important differences between layers of sediment and significant changes in community composition with zone (canyon vs slope), depth, and season. We compared the information obtained from metabarcoding DNA and RNA and found more total MOTUs and more MOTUs per sample with DNA (ca. 20% and 40% increase, respectively). Both datasets showed overall similar spatial trends, but most groups had higher MOTU richness with the DNA template, while others, such as nematodes, were more diverse in the RNA dataset. We provide metabarcoding protocols and guidelines for biomonitoring of these key communities in order to generate information applicable to management efforts.

## Introduction

The use of environmental DNA (eDNA) is revolutionizing the way we assess biodiversity and has the potential to change practices and policies in management and conservation (Bohmann et al., 2014; Kelly et al., 2014; Handley, 2015; Creer et al., 2016). The era of eDNA was initiated in prokaryote microbiology (e.g., Sogin et al., 2006; Roesch et al., 2007) and subsequently expanded to the study of eukaryote diversity, of both micro- and macro-organisms (e.g., Bik et al., 2012a; Fonseca et al., 2014; Leray & Knowlton, 2015; Leray & Knowlton, 2016; Thomsen & Willerslev, 2015; Brannock & Halanych, 2015; Brannock et al., 2016).

The detection of biodiversity using genetic tags obtained from eDNA (metabarcoding, Taberlet et al., 2012a) provides a fast and reliable method for monitoring biodiversity (Ji et al., 2013; Dafforn et al., 2014; Leray & Knowlton, 2016). Although several technical and methodological challenges remain, such as those related to contamination, primer biases, sequencing artefacts, delineation of taxonomic units, or databases’ depth (Thomsen & Willerslev, 2015; Carugati et al., 2015; Pawlowski, Lejzerowicz & Esling, 2014a; Leray & Knowlton, 2016; Wangensteen & Turon, 2016), metabarcoding has important advantages relative to morphology-based studies. Among them, speed, cost per sample, coverage, independence of taxonomic expertise, and ability to detect unsampled species that leave DNA in the environment (Rees et al., 2014). Recent studies combining molecular and traditional assessments confirm the potential of metabarcoding (e.g.,  Pawlowski et al., 2014b; Dafforn et al., 2014; Zimmermann et al., 2015; Cowart et al., 2015; Lejzerowicz et al., 2015; Pearman et al., 2016). In management and conservation, this approach allows moving away from the traditional use of one or a few indicator species (often biased towards emblematic or apparent species) towards a direct exhaustive biodiversity assessment (Ji et al., 2013; Handley, 2015). The field is ripe for the move from descriptive, academic objectives to applied biomonitoring goals (Bohmann et al., 2014; Pawlowski et al., 2016; Bucklin et al., 2016).

If metabarcoding is to become an efficient tool to generate management and conservation guidelines (Creer et al., 2016), it has to show its applicability in routine follow-up of communities over space and time, unravelling trends linked to environmental variables or to human impacts. In marine environments, some metabarcoding studies have analysed ecological patterns looking for drivers of biodiversity (e.g., Dafforn et al., 2014; Fonseca et al., 2010; Fonseca et al., 2014; Chariton et al., 2015; Lallias et al., 2015; Brannock et al., 2016), while others have explored assessment of human impacts or early surveillance of introduced species (e.g., Bik et al., 2012b; Brannock et al., 2014; Pawlowski et al., 2014b; Lejzerowicz et al., 2015; Pochon et al., 2015; Zaiko et al., 2016).

Few studies have used simultaneously eDNA and eRNA in metabarcoding of marine eukaryotes, and only one to our knowledge involved metazoans (Lejzerowicz et al., 2015). RNA may be less biased than DNA (Not et al., 2009) and better reflect environmental changes (Pawlowski et al., 2014b), as it recovers preferentially the active fraction of the biomass. In addition, the persistence of eDNA may buffer intersample variability and artificially reduce *β*-diversity estimates (Lejzerowicz et al., 2015).

Sedimentary bottoms, given the extraordinary diversity and small size of most of their inhabitants, are particularly difficult to study with standard morphological methods. Not surprisingly, sediment communities are prominent (along with plankton assemblages) in metabarcoding studies of the marine realm to date (reviewed in Sinniger et al., 2016). However, few of these works have analysed eukaryote diversity in deep-sea sediments (Lecroq et al., 2011; Pawlowski et al., 2011; Bik et al., 2012c; Lejzerowicz et al., 2013; Orsi, Biddle & Edgcomb, 2013; Sinniger et al., 2016), and none to our knowledge has investigated the temporal component of variability in deep-sea assemblages.

Deep-sea ecosystems are nowadays a research frontier where the use of novel technologies is uncovering hidden biodiversity, revealing unknown habitat complexity and shedding light on interactions and adaptations (reviewed in Danovaro, Snelgrove & Tyler, 2014). The deep-sea bottoms occupy more than 65% of the planet surface and constitute the largest biome on Earth (Thistle, 2003; Ramirez-Llodra et al., 2011) yet only a small fraction of the diversity present in them is known (Snelgrove, 1999; Danovaro et al., 2010). Deep-sea habitats provide essential ecosystem services in nutrient cycling and biogeochemical processes (Gage & Tyler, 1991; Ramirez-Llodra et al., 2010). Given the threats that this crucial habitat faces nowadays as a result of human activities (Ramirez-Llodra et al., 2010; Ramirez-Llodra et al., 2011; Pusceddu et al., 2014), it is imperative to develop monitoring programs based on the most effective biodiversity assessment techniques available.

The Mediterranean Sea provides a unique setting for the study of the deep-sea habitats. Although it represents only 0.7% of the area of the global ocean, it contains 8.85% of the known deep submarine canyons (Canals et al., 2013). In particular, the Northwestern Mediterranean coasts are carved by a system of canyons where physical and biogeochemical factors regulate the deep-sea communities and their productivity (Canals et al., 2013; Danovaro et al., 2009; Danovaro et al., 2010; Lopez-Fernandez et al., 2013; Pusceddu et al., 2010). These canyons and adjacent margins are biodiversity hotspots (Danovaro et al., 2010; Ramirez-Llodra et al., 2009), have important fishing grounds associated (Sardà et al., 2004; Ramirez-Llodra et al., 2009), and face significant threats from human activities (Palanques et al., 2006; Martín et al., 2008; Puig et al., 2012; Canals et al., 2013).

Our study focuses on one of the main canyons in the area, the Blanes Canyon (Lastras et al., 2011) and its adjacent slopes. We analysed spatial (along a depth gradient) and temporal (at two seasons) patterns and compared the information obtained from eDNA and eRNA. Our final goal was to develop and test protocols and guidelines for the use of metabarcoding in biomonitoring of these key communities, thus generating information applicable to management and conservation efforts.

## Material and Methods

### Sampling

**Figure 1:**
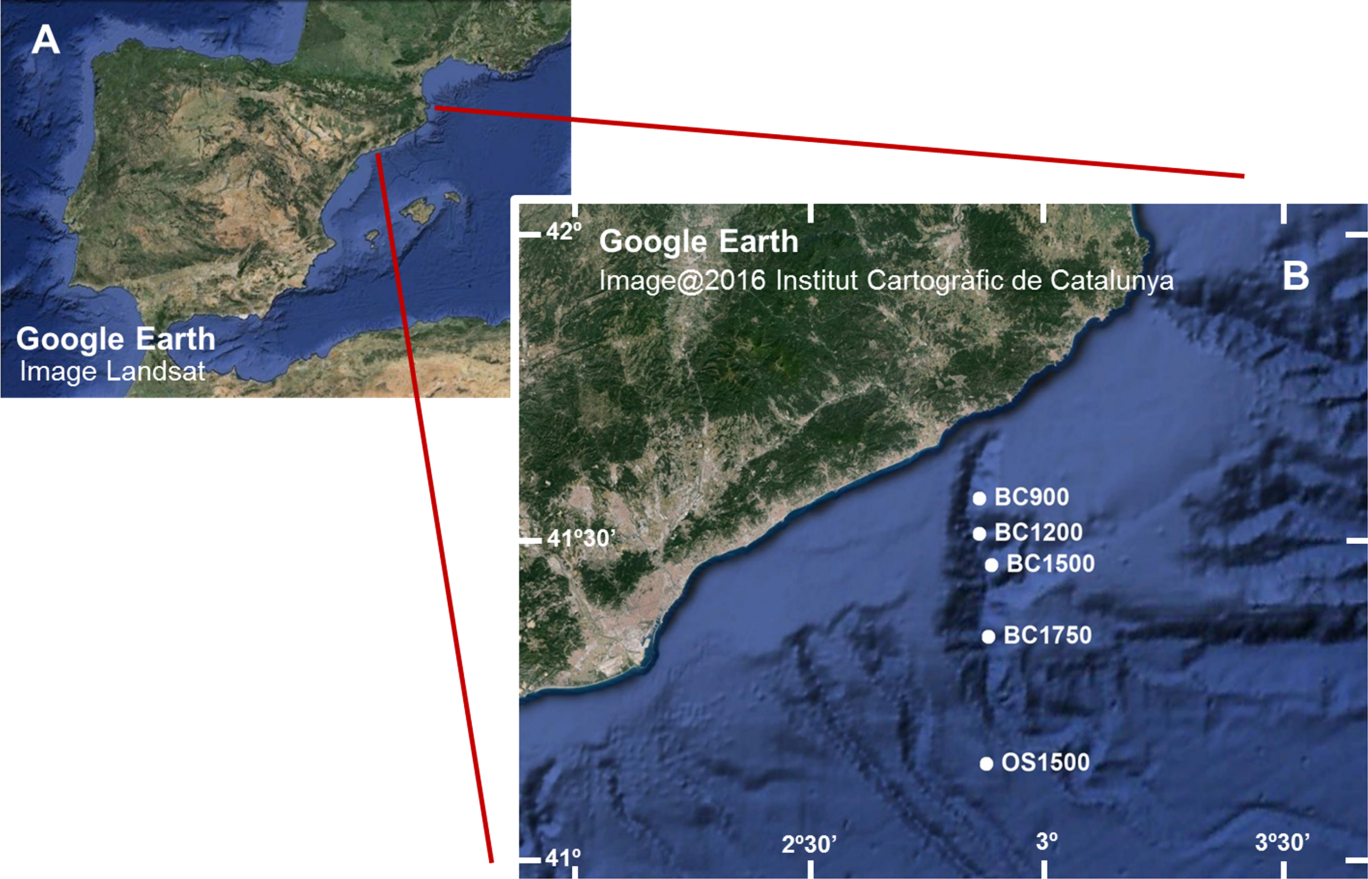
(A) Image of the Iberian Peninsula (Google Earth, Landsat) and (B) enlargement showing the Blanes Canyon with indication of the sampling stations (Google Earth, Institut Cartogràfic de Catalunya). Codes as in Table 1.

We obtained sediment samples from the Blanes Canyon (Western Mediterranean Sea, NE Iberian Peninsula) and the adjacent open slope (Fig. 1 and Table 1). The canyon is over 180 km long and its deeply incised upper part extends in a N-S direction. The canyon becomes flat-floored and meandering at the base of the slope, and the lower canyon turns to a W-E course, ending at ca. 2,600 m. Particle fluxes in the canyon are three times higher than on the adjacent open slope, and the discharge of the Tordera River at the head of the canyon determines pulses of descending particulate matter (Lastras et al., 2011; Jorda et al., 2013).

**Table 1:** List and characteristics of the sampling localities of the DOSMARES II and III cruises where samples for this study were collected.

Zone	Date	Locality	Depth (m)	Lat N	Long E
DOSMARES II Cruise					
Blanes Canyon (BC)	07/Oct/2012	BC900	874	41°34′17″	02°50′52″
	07/Oct/2012		890	41°34′14″	02°50′47″
	07/Oct/2012		852	41°34′17″	02°50′35″
	07/Oct/2012	BC1200	1,232	41°30′43″	02°50′46″
	07/Oct/2012		1,194	41°30′44″	02°50′35″
	08/Oct/2012		1,248	41°30′44″	02°50′54″
	08/Oct/2012	BC1500	1,450	41°27′31″	02°52′57″
	08/Oct/2012		1,463	41°27′38″	02°52′46″
	08/Oct/2012		1,457	41°27′29″	02°52′58″
	11/Oct/2012	BC1750	1,746	41°21′16″	02°52′13″
	11/Oct/2012		1,751	41°21′20″	02°52′13″
	11/Oct/2012		1,727	41°21′38″	02°52′15″
Blanes Open Slope (OS)	12/Oct/2012	OS1500	1,454	41°08′42″	02°53′33″
	12/Oct/2012		1,451	41°08′37″	02°53′32″
	12/Oct/2012		1,480	41°08′30″	02°53′48″

Zone	Date	Station	Depth (m)	Lat N	Long E
DOS MARES III Cruise					
Blanes Canyon (BC)	05/Apr/2013	BC900	870	41°34′19″	02°50′59″
	05/Apr/2013		903	41°34′19″	02°50′56″
	05/Apr/2013		899	41°34′19″	02°50′53″
	06/Apr/2013	BC1200	1,227	41°31′01″	02°51′03″
	06/Apr/2013		1258	41°30′52″	02°50′50″
	10/Apr/2013		1,234	41°30′46″	02°50′54″
	10/Apr/2013	BC1500	1,483	42°27′15″	02°52′57″
	23/Apr/2013		1,516	41°27′03″	02°52′53″
	24/Apr/2013		1,520	41°27′02”	02°52′50″
	23/Apr/2013	BC1750	1,730	41°21′14″	02°51′54″
	23/Apr/2013		1,785	41°21′14″	02°52′17″
	23/Apr/2013		1,830	41°21′06″	02°52′20″
Blanes Open Slope (OS)	22/Apr/2013	OS1500	1,507	41°08′28″	02°53′46″
	23/Apr/2013		1,480	41°08′23″	02°53′33″
	23/Apr/2013		1,456	41°08′18″	02°53′41″

The sampling was done with a multicorer device (holding six corers 10 cm in diameter) from the *R/V García del Cid* of the Spanish Research Council. The same points were sampled in two cruises: autumn 2012 (DOSMARES II) and spring 2013 (DOSMARES III) (Table 1). Samples were taken at depths of 900 m, 1,200 m, 1,500 m and 1,750 m inside the canyon, and 1,500 m in the open slope. Three multicorers, less than 600 m apart, were deployed at each depth and subsampled by taking one mini-corer (3.6 cm diameter, 5 cm thickness) from one of the larger sediment corers. The mini-corer samples were then divided into three layers (first cm, second cm and third to fifth cm) and preserved in absolute ethanol. A total of 90 samples, 45 per season (5 stations × 3 multicorers × 3 layers), were obtained and used for DNA extraction. For the RNA-DNA comparison, a second mini-corer was taken during the autumn cruise from the same multicorers as in the DNA study, corresponding to the depths of 1,200 m, 1,500 m and 1,750 m in the Blanes Canyon and 1,500 m in the open slope. The sediment was divided into layers as described above (totalling 36 samples: 4 stations × 3 multicorers × 3 layers) and frozen in liquid nitrogen to preserve RNA.

### DNA and RNA extraction, amplification and sequencing

For DNA extraction, 10 g of sediment of each sample were processed with the PowerMax^®^ Soil DNA Isolation Kit (MO BIO Laboratories, Inc.). For the RNA-DNA comparison, 2 g of sediment of the 36 additional samples were processed with the RNA PowerSoil^®^ Total RNA Isolation Kit, using the modification of the protocol suggested by the manufacturer for salty sediments. For co-isolation of DNA from the same samples from which RNA was obtained, the RNA PowerSoil^®^ DNA Elution Accessory Kit was used on the same column from which the RNA had been eluted. The extracts from the RNA isolation were treated with DNase Amplification Grade (Invitrogen) to eliminate any remaining DNA. The cDNA synthesis was carried out using the SuperScript^®^ VILO™cDNA Synthesis Kit (Invitrogen). All downstream processes were identical for DNA and RNA extracts.

The genetic marker used was a hypervariable fragment of the v7 region of the 18S rRNA gene (Hadziavdic et al., 2014). The universal primers 18S_allshorts (Forward 5′-TTTGTCTGSTTAATTSCG-3′ and Reverse 5′-TCACAGACCTGTTATTGC-3′ were used (see Guardiola et al., 2015 for details on these primers and their suitability). The primers span the region comprised between positions 1,301 and 1,436 (using *Saccharomyces cerevisiae* 18S rRNA gene as template, Hadziavdic et al., 2014). Amplification was performed in a total volume of 30 µl with 0.24 µl of AmpliTaq^®^ Gold DNA polymerase (Applied Biosystems) 5 U/µl, 1.2 µl of 5 µM of forward and reverse primers mix, 3 µl of buffer 10×3 µl of MgCl_2_, 2.4 µl dNTP (2.5 mM each), 0.24 µl of BSA (20 mg/ml) and 3 µl of DNA template. The PCR conditions consisted in a first denaturation step of 10 min at 95  °C and then 45 cycles of denaturation at 95 °C for 30 s, hybridisation at 45 °C for 30 s and elongation at 72 °C for 30 s. Tags of 8 base pairs were added to the forward and reverse primers to uniquely label each sample (the same tag was used at both ends). The tags were created with the program OligoTag (Boyer et al., 2016) and had at least 3 different base pairs between each other. Along with the samples, 4 negative controls with ultrapure water (Milli-Q System) and 4 blanks with PCR mixture without DNA template were run. Library preparation and sequencing was performed by FASTERIS (Plan-les-Ouates, Switzerland; https://www.fasteris.com/dna/) using a complete run on an Illumina MiSeq platform (2 × 150 bp paired-ends).

### Read filtering and taxon assignment

The sequence reads were analysed using the OBITools software (Boyer et al., 2016). The paired-ends of each sequence were assembled and those with an alignment score <40 were discarded. Sequences of non-suitable length (<75 bp excluding primers and tags) were removed. Sample tags were used to assign reads to samples. As the tags were identical at both ends, any inter-sample PCR chimeras were eliminated at this step. Strictly identical sequences were dereplicated and assigned a count number per sample. Singleton sequences (i.e., sum of counts = 1) were eliminated.

A preliminary cleaning step was carried out to remove highly divergent sequences that would interfere with the Bayesian clustering procedure (see below). First, a reference database was built with ecoPCR (Ficetola et al., 2010) for the 18S fragment from the release 117 of the EMBL database. Then, sequences with less than 0.8 similarity with a sequence in this reference database were eliminated. This “cleaning by similarity” step effectively removed most sequences with length >120 bp probably corresponding to chimeras, sequencing errors, or nonspecific amplifications, while retaining those that likely correspond to true organisms (Fig. S1). The retained sequences were checked with UCHIME (both *de novo* and against the reference database, Edgar et al., 2011) and no remaining chimeric sequences were found.

The sequences were clustered into molecular operational taxonomic units (MOTUs) using the Bayesian clustering algorithm implemented in CROP (Clustering 16S rRNA for OTU Prediction, Hao, Jiang & Chen, 2011). This method uses a Gaussian mixture model to infer the optimal clustering of the data without setting a single fixed similarity threshold for all clusters. When dealing with diverse communities, it is unlikely that a single threshold suits all groups, and flexible threshold methods are preferable (Hao, Jiang & Chen, 2011; Mahé et al., 2014; Brown et al., 2015; Wangensteen & Turon, 2016). We set the model parameters to I = 0.3 and u = 0.5, which correspond to an initial clustering level at 99% similarity (https://code.google.com/p/crop-tingchenlab/#Parameters). Clusters were then refined iteratively until the results satisfied the termination conditions (Hao, Jiang & Chen, 2011). The 99% initial value corresponds to the “stringent clustering” of Bik et al. (2012c), but the final similarity degree of a given MOTU can be higher or lower than the initial value depending on the natural organization of sequences in multidimensional space. The most abundant sequence in each MOTU was then used as the representative of the cluster. The delineation of MOTUs tries to match as close as possible the true species-level diversity. However, an exploration of some well-represented metazoan groups in our reference database showed instances of different species sharing the same sequence for the fragment analysed. Therefore, our results are likely conservative in terms of biodiversity detected; but this was preferable in our view than to have an inflation of MOTUs.

MOTUs were taxonomically assigned using the ecotag program (Boyer et al., 2016), queried with the representative sequence of each MOTU. The program searches the best hit in the reference database and builds a set with all the sequences in the database that are as similar or more to the best hit as the query sequence is. The taxon that is the most recent common ancestor to all these sequences in the NCBI taxonomy database is then assigned to the MOTU. As a result, the taxonomic category assigned varies depending on the similarity of the query sequences and the density of the database. The use of the ecotag procedure allowed us to robustly assign MOTUs even if the minimal similarity threshold chosen (0.8) was relatively low (MOTUs whose best hit was in the lower range of similarities were in general assigned only at high taxonomic ranks). The MOTUs were then classified following the major Super-Groups of eukaryotes (Guillou et al., 2013), with one exception: Opisthokonta were split into Metazoa, Fungi and other Opisthokonta. MOTUs that could not be identified at least at the Super-Group level were eliminated. Metazoan MOTUs were further classified into Phyla for additional analyses, and those that could not be assigned a Phylum were excluded from the analyses within Metazoa.

Once the taxa list was acquired, further filtering was performed to refine the dataset. For each MOTU the counts per sample were ordered from lowest to highest and those corresponding to a cumulative frequency of less than 0.03 were set to 0 (this step aimed at eliminating possible cross-sample contamination during the library preparation step). Second, MOTUs present in the negative controls and blanks after the previous step were removed. Finally, using the Taxon Match Tool of WoRMS (http://www.marinespecies.org/aphia.php?p=match), non-marine organisms were removed (these could represent contaminations or DNA of continental origin present in the sediment). The raw assembled sequences, once demultiplexed, as well as the final dataset of MOTUs, with best match hits, taxonomic rank assigned, taxon names and number of reads per sample have been deposited in the Dryad Digital Repository (http://dx.doi.org/10.5061/dryad.4kn05).

### Data analysis

The dataset of MOTUs was divided in subsets for the different analyses. First, a spatio-temporal study using DNA included the 90 samples (45 for autumn and 45 for spring) obtained from the Blanes Canyon and the adjacent open slope. Second, the RNA-DNA comparison was performed using the 36 additional samples of the autumn collection trip from which RNA and DNA had been co-extracted.

Rarefaction curves were obtained with function *rarecurve* of the vegan 2.0-7 package (Oksanen et al., 2016) in R 3.1.2. Venn diagrams for the number of MOTUs found in each of the three sediment layers were constructed with EulerAPE 3.0.0 (Micallef & Rodgers, 2014), which generates proportional diagrams using ellipses instead of circles.

Permutational analyses of variance were performed with the Windows PERMANOVA module (Anderson, Gorley & Clarke, 2008) incorporated in the Primer v6 statistical package (Clarke & Gorley, 2006). Similarity based on presence/absence data (Jaccard index) was used to assess significance of relevant factors. For the spatio-temporal study, comparisons were made between layers, zones (Blanes Canyon vs open slope), depths, and seasons. For the RNA-DNA comparison, the effects of nucleic acid type (RNA/DNA) and zone were tested. Permutational pair-wise tests were done for all significant factors and their *p* values corrected for multiple comparisons with the Benjamini-Yekutieli FDR correction (Narum, 2006). Tests of multivariate dispersions (PERMDISP) were run for significant factors to determine whether significant values in PERMANOVA were a result of different multivariate mean or different heterogeneity (spread) of the groups.

Reduced-space graphical representation of the data was obtained by non-metric multidimensional scaling (nMDS) ordinations. These analyses were performed with the *metanmds* function of the package vegan (Oksanen et al., 2016) with 500 random starts. The nMDS ordinations were obtained using distance matrices based on the Jaccard index. Function *envfit* of vegan was used to correlate depth with the ordinations of the samples inside the canyon and to plot the corresponding best-fit vector.

## Results

After paired-end assembly, quality and length filtering, and elimination of singletons, the global dataset (all samples pooled) consisted of 11,348,001 reads corresponding to 235,484 unique sequences. Elimination of divergent sequences (less than 80% similarity to sequences in the database) left 9,691,911 reads and 175,570 sequences.

The CROP procedure found 10,073 clusters. Blanks and negative controls had negligible numbers of reads (mean of 177.8). After further filtering based on cumulative frequencies (see methods), elimination of MOTUs that could not be assigned to Super-Group or lower rank, and non-marine organisms, the final dataset consisted of 5,569 MOTUs with a total of 5,728,801 reads (Table S1). Rarefaction curves (Fig. S2) showed that a plateau in the number of MOTUs was achieved in general at ca. 20,000 reads which, considering that the mean number of reads obtained per sample was 35,362, indicated an overall sequencing depth adequate to capture the number of MOTUs present. These MOTUs were distributed in 11 Super-Groups (Table S1), with Metazoa being the best represented (1,881 MOTUs in total), followed by Alveolata (1,480 MOTUs) and Rhizaria (74 MOTUs). 20 metazoan Phyla were identified (Table S1), of which Nematoda was the most diverse with 747 MOTUs. Arthropoda and Annelida were second and third with 255 and 149 MOTUs, respectively. The taxonomic ranks at which MOTUs were assigned by the ecotag procedure for metazoans in general and for the three most diverse metazoan Phyla are presented in Fig. S3. Proportionally more annelids could be assigned to low taxonomic categories (species and Genus) than nematodes or arthropods. The number of taxa of the different taxonomic categories identified is represented for metazoans in Fig. S4. A higher number of genera than species was found, reflecting the fact that many MOTUs could not be assigned to a particular species

### Spatio-temporal patterns

**Figure 2:**
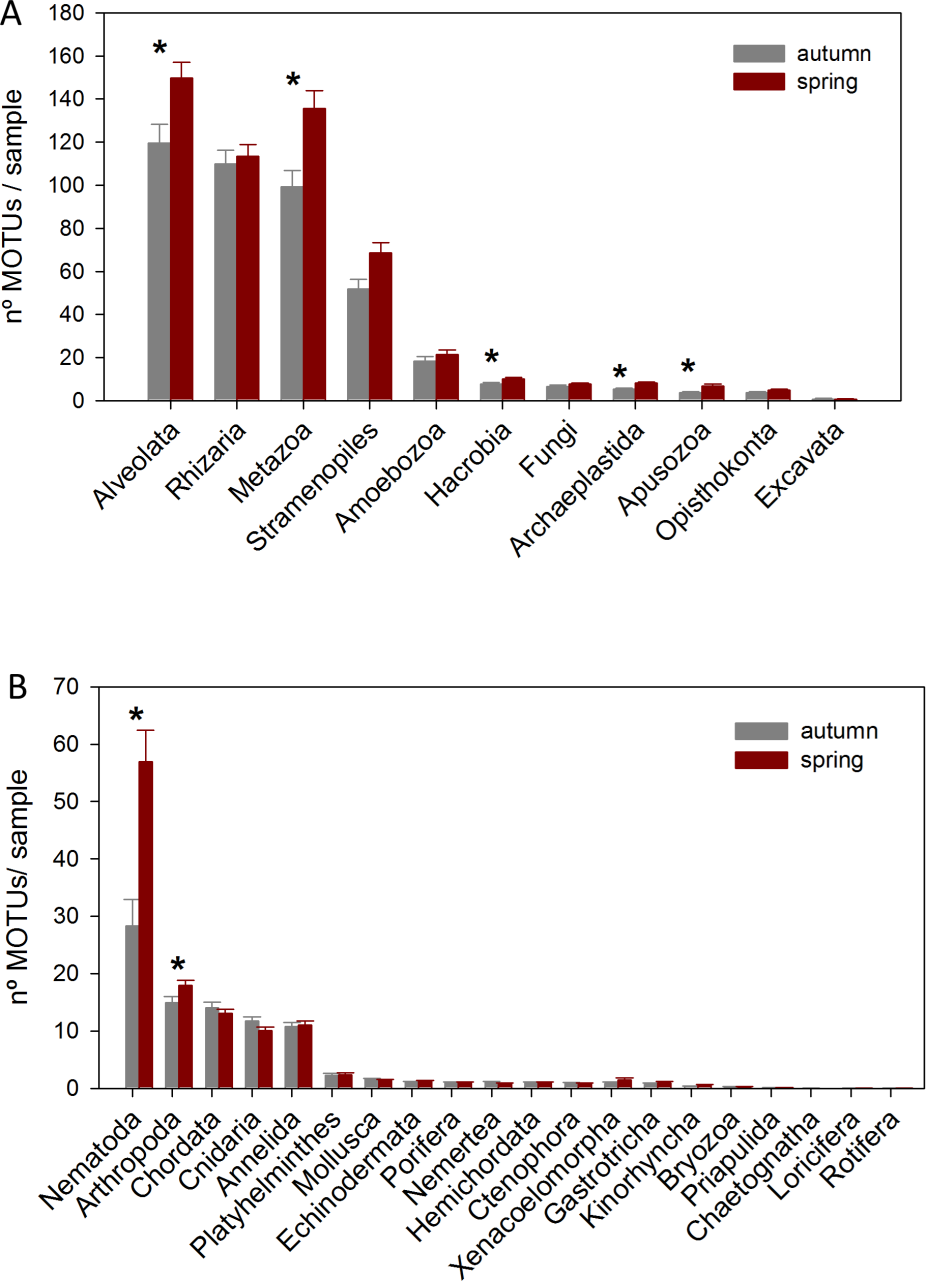
Mean number of MOTUs per sample for the different Super-Groups (A) and metazoan Phyla (B) considered at the two seasons sampled (*: significant differences between seasons assessed by *t*-tests). Bars are standard errors.

The DNA samples (45 in autumn and 45 in spring) yielded a total of 4,953 MOTUs. The pattern of MOTU richness per sample for the different Super-Groups showed some differences in autumn and spring (Fig. 2A). Alveolata were the most diverse, followed by Rhizaria in autumn and Metazoa in spring. Overall, slightly more MOTUs were obtained in spring than in autumn (4,125 vs 3,968, respectively, Table S1). Statistical comparisons (*t*-tests) between seasons showed a significantly higher number of MOTUs per sample for Alveolata, Metazoa, Hacrobia, Archaeplastida and Apusozoa in spring.

**Figure 3:**
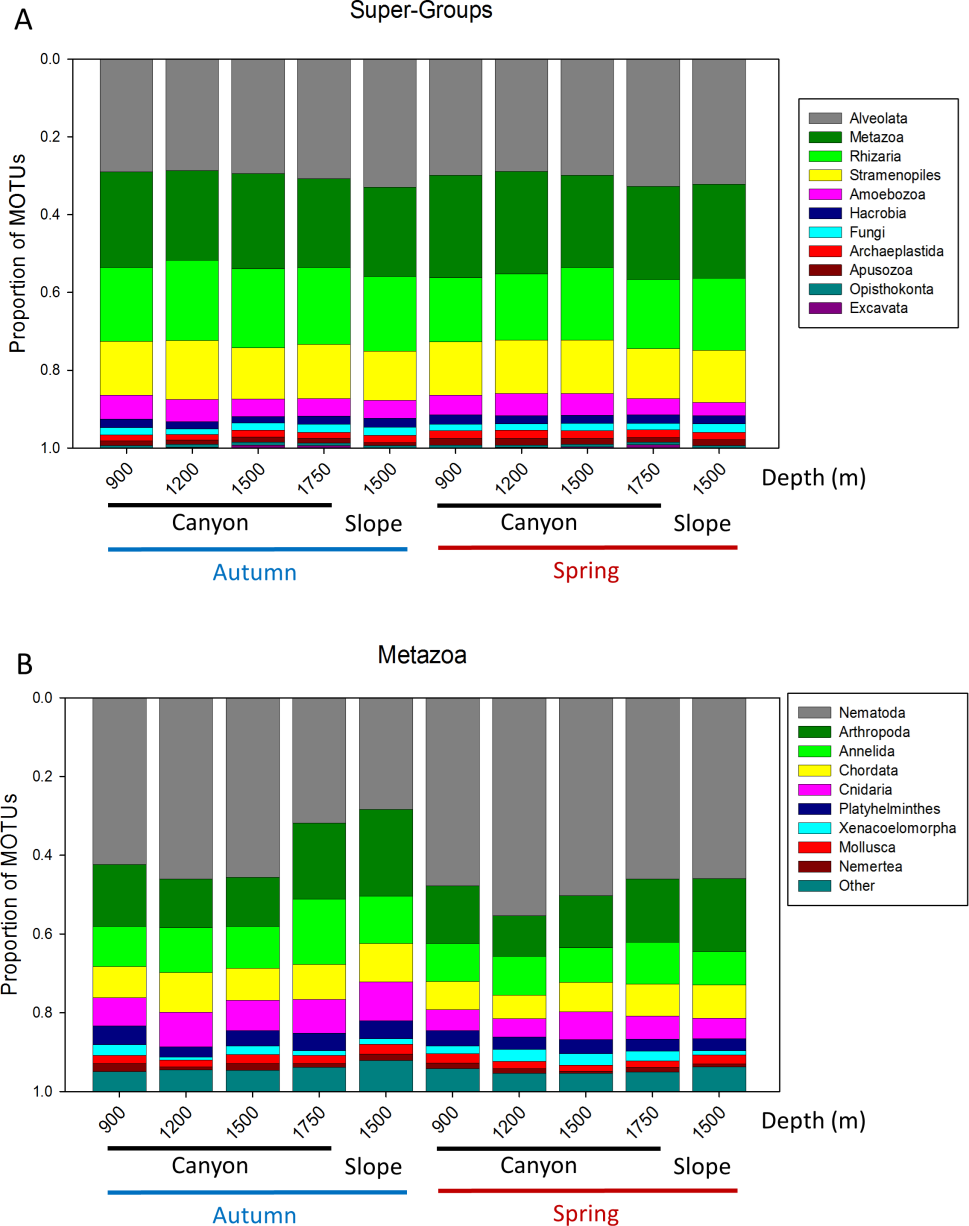
Proportion of MOTUs of the different Super-Groups (A) and metazoan Phyla (B) per sampling station and season.

A more detailed study (by Phylum) was carried out on the metazoans (Fig. 2B). Nematoda was the most MOTU-rich Phylum, followed by Arthropoda, Chordata, Cnidaria and Annelida. All other groups had a relatively minor contribution. Marked temporal differences were found for Nematoda, which doubled their richness per sample in spring, while Arthropoda also had significantly more MOTUs per sample in spring (*t*-tests, Fig. 2B). The proportion of MOTUs of the different Super-Groups and metazoan Phyla as per sampling locality is shown in Fig. 3.

When analysing the spatial structure in terms of sediment layers, the most superficial layer (layer A, first cm of sediment) was the most diverse (3,821 MOTUs in total), followed by the intermediate (B, second cm) and deep (C, third to fifth cm) layers (3,441 and 2,848 MOTUs, respectively) (Fig. S5). The number of MOTUs found in a single layer of sediment also decreased with layer depth: 902 MOTUs were found exclusively in layer A; 461 MOTUs in layer B, and 362 MOTUs in layer C. Overall, 1,929 MOTUs were shared by the three layers.

**Figure 4:**
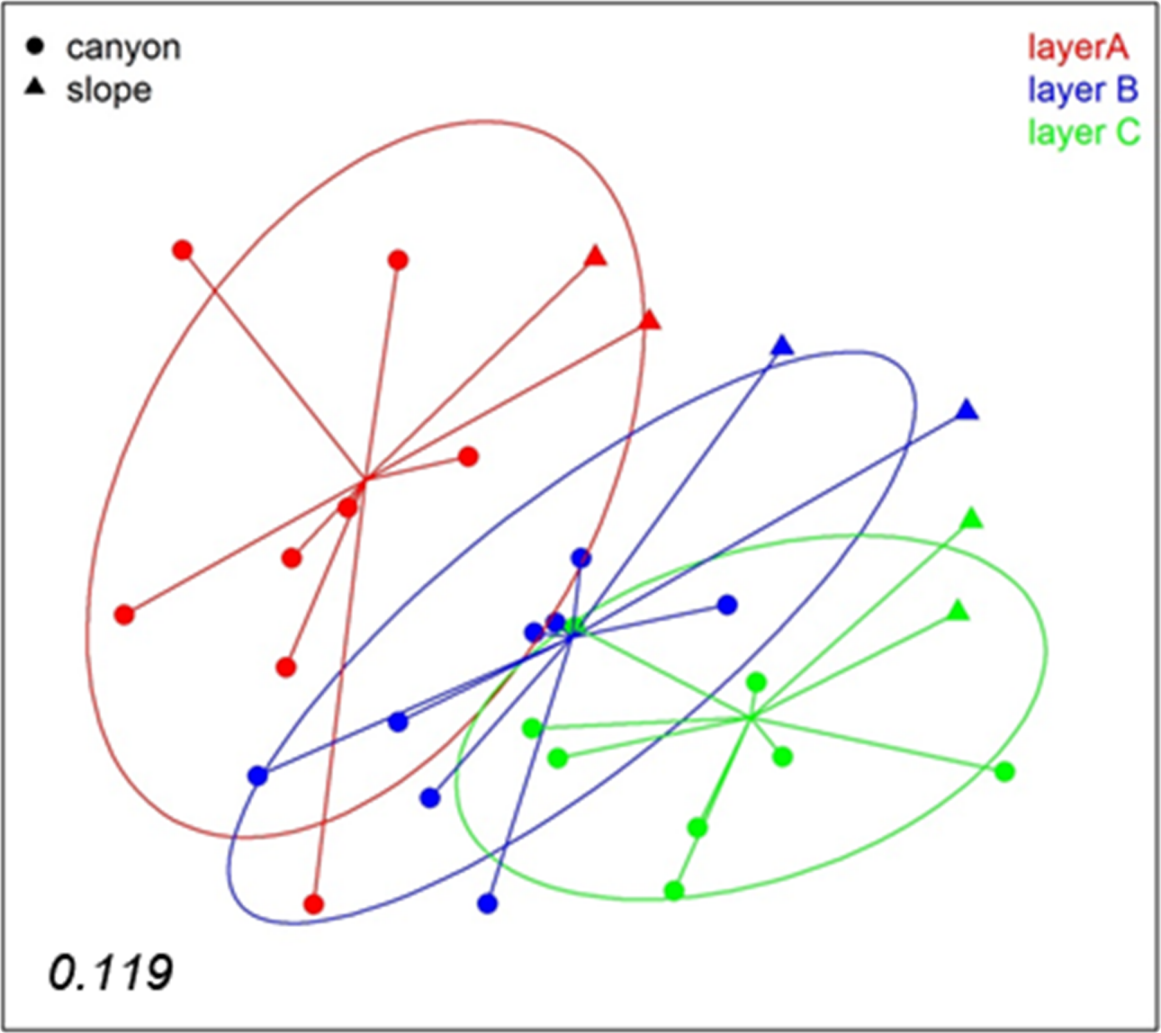
nMDS representation of the samples by layers (the three replicates per locality pooled). The number in the lower left corner indicates the stress value of the final configuration.

A nMDS analysis based on presence/absence data (Jaccard index) for the different layers of sediment (the three replicate samples of each locality pooled) revealed differences between layers (Fig. 4), which tended to group following a gradient in layer depth. The samples from the open slope followed the same pattern as those from the canyon, but were somewhat set apart. The stress value (0.119) suggests an adequate 2-dimensional picture of sample distribution. A PERMANOVA analysis of the factors layer (fixed) and locality (random), with corer as a blocking factor nested in locality, showed a significant layer effect in community structure (Table S2). Locality, corer, and the interaction between layer and locality were also highly significant, an effect not due to differences in dispersion levels (non-significant PERMDISP tests, Table S2). Pairwise tests revealed that layer A was different from either layer B or C, but the latter two did not differ significantly (Table S2).

**Figure 5:**
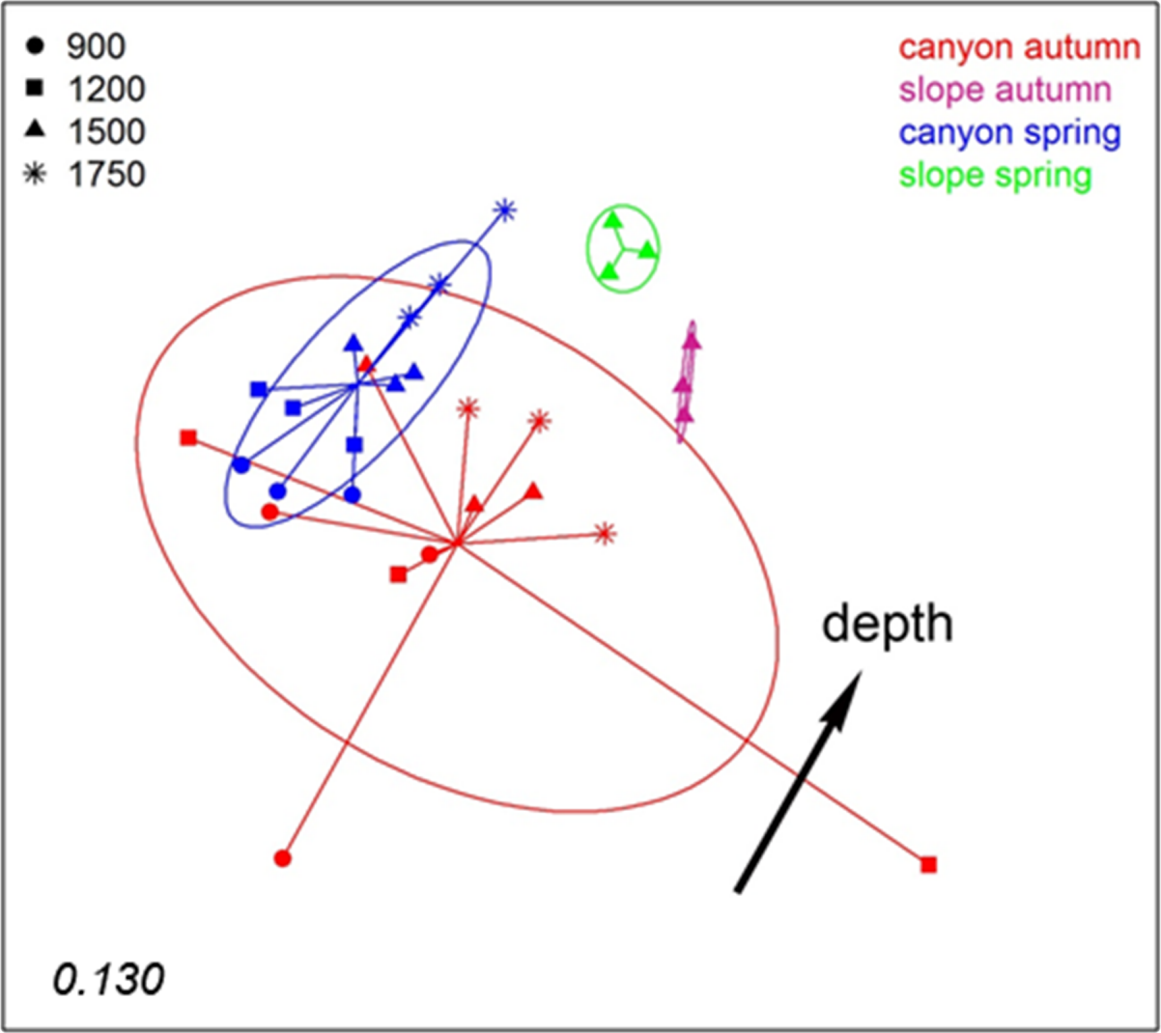
nMDS representation of the samples of the spatio-temporal study (the three layers per sample pooled). The number in the lower left corner indicates the stress value of the final configuration. The fitted vector depth considering only the samples in the canyon is added (displaced from the center for clarity).

To analyse spatio-temporal patterns between localities, the three layers of each sample were merged. A nMDS ordination showed a clear distinction between the Blanes Canyon and the adjacent open slope (Fig. 5). The centroids of the two seasons appeared also separated, but with overlap of the inertia ellipses in the canyon. The autumn samples in the canyon showed a markedly higher dispersion than the spring samples, indicating higher heterogeneity in community composition (Fig. 5). Indeed, the values of the Jaccard similarity index between samples in the canyon were significantly higher (*t*-test) in spring that in autumn (42.09 ± 0.66% vs 36.50 ± 0.87%, mean ± SE, respectively). At each season, a depth gradient within the canyon was apparent (Fig. 5). The correlation of depth with the ordination obtained (calculated only with the canyon samples) was highly significant (*r*^2^ = 0.696, *p* < 0.001).

PERMANOVA confirmed the significant effect of zone (canyon vs open slope) and season on community structure (Table S3), while the interaction was not significant. To avoid the confounding effect of depth, in this analysis only samples at equivalent depth in the canyon and the slope (1,500 m) were included. In agreement with the spatial configuration detected in nMDS, the two seasons sampled showed significant differences in dispersion (PERMDISP results, Table S3). A further PERMANOVA was performed only with samples from inside the canyon to test the effect of season and depth, which were both significant, while their interaction was not (Table S4). The PERMDISP test showed again differences in dispersion of the samples for the season factor. Pairwise comparisons between depths revealed significant differences when comparing samples separated by two or more depth levels (Table S4).

**Figure 6:**
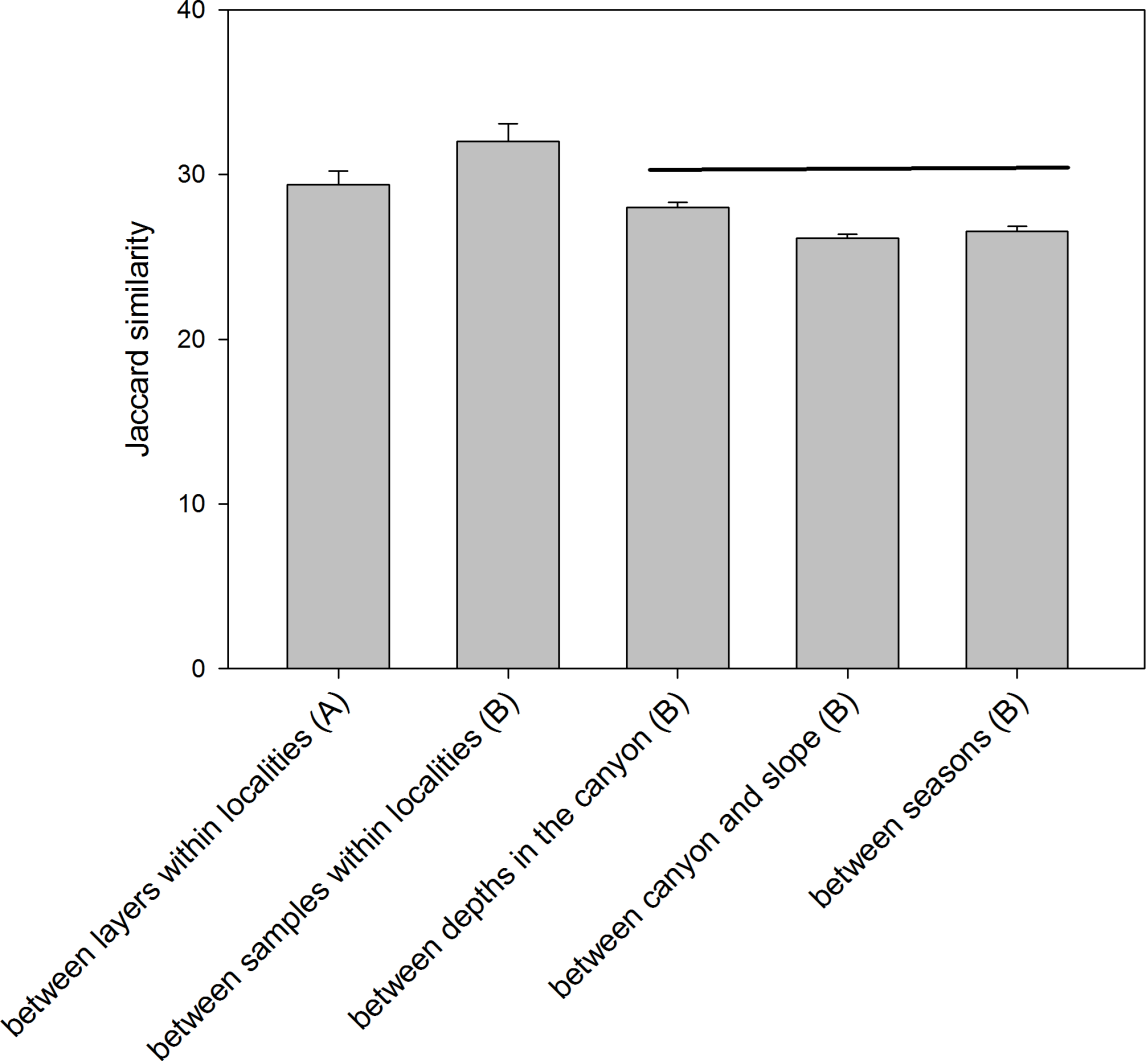
Mean values of similarity based on presence/absence of MOTUs (Jaccard index) among different sample groups. Bars are standard errors. Horizontal lines join values not significantly different in an ANOVA. (A): samples pooled within localities; (B): layers pooled within samples.

The comparison of the similarity (Jaccard index) among different combinations of samples (Fig. 6) showed an overall high differentiation (all values below 35%). The similarity is highest when comparing layers (the three replicate samples pooled) and when comparing samples (layers pooled) within each locality (29.39 ± 0.82% and 32.00 ± 1.07%, respectively). The comparisons between depths (in the canyon), between zones (canyon and open slope) and between seasons (autumn and spring) had significantly lower values (26–28%) and were not significantly different among them (ANOVA and Student-Newman-Keuls *post-hoc* comparisons, Fig. 6).

### RNA-DNA comparison

We analysed the RNA and DNA co-isolated from the same sediment from 3 localities inside the canyon and one in the slope in the autumn sampling. We obtained a total of 4,151 MOTUs, 3,542 from the DNA and 2,931 from the RNA, with a similar number of reads (524,439 and 518,905, respectively, Table S1). 609 MOTUs that were found with RNA failed to be detected in the DNA, while 1,220 MOTUs appeared only in the DNA samples, of which 425 were Alveolata. Not only was the total number of MOTUs 20.8% higher with DNA, but there was also a 41.9% higher number of MOTUs per sample (481.9 ± 37.2 and 339.5 ± 38.6, for DNA and RNA, respectively). Concerning the number of taxa of the different categories recovered, 37–45% more taxa were assigned with DNA at the lower taxonomic categories (Family, Genus, and species, Fig. S6). For the most diverse metazoan Phylum (Nematoda), however, the number of taxa recovered for the different categories was similar with DNA and RNA, even slightly higher with the latter at the lower categories (Fig. S6).

**Figure 7:**
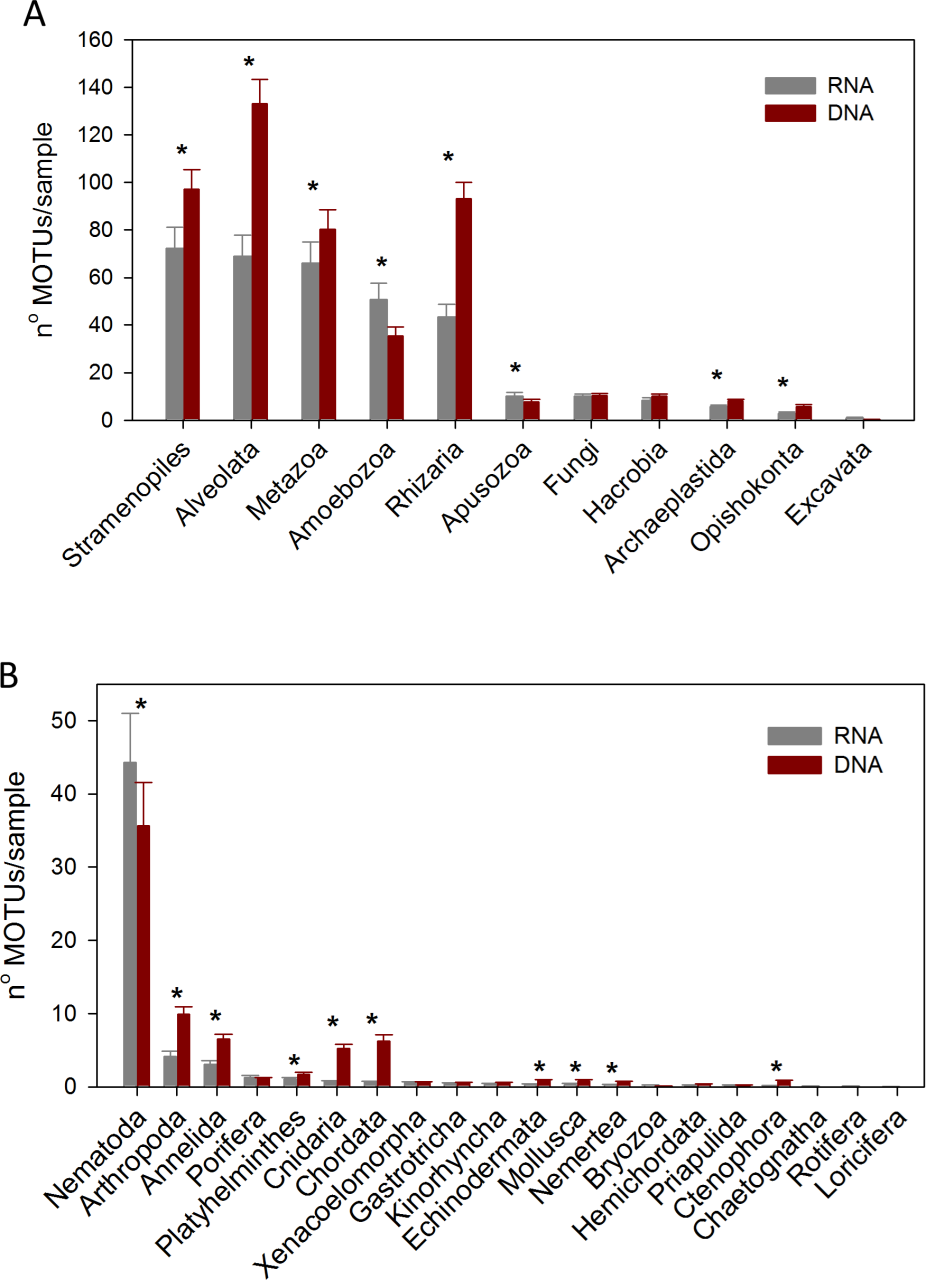
Mean number of Super-Group (A) and metazoan (B) MOTUs per sample for the RNA and DNA datasets (*: significant differences between methods assessed by paired *t*-tests). Bars are standard errors.

Considering the number of MOTUs per sample of the different Super-Groups (Fig. 7A), the most diverse group found with RNA was Stramenopiles, followed by Alveolata and Metazoa. For DNA, the ranking was Alveolata, Stramenopiles and Rhizaria. In all these taxa the number of MOTUs found per sample was significantly higher (paired *t*-tests) in the DNA than in the RNA extracts. The same happened in Archaeplastida and Opisthokonta (excluding Metazoa and Fungi). Only in the case of Amoebozoa and Apusozoa did the number of MOTUs recovered from RNA exceed significantly those from DNA.

In the analysis of the metazoan Phyla (Fig. 7B), Nematoda appeared as the most diverse group for both DNA and RNA extractions, but there were significantly more nematode MOTUs per sample with RNA. On the contrary, significantly more MOTUs per sample were obtained with DNA for 9 metazoan Phyla (Arthropoda, Annelida, Platyhelminthes, Cnidaria, Chordata, Echinodermata, Mollusca, Nemertea, Ctenophora).

**Figure 8:**
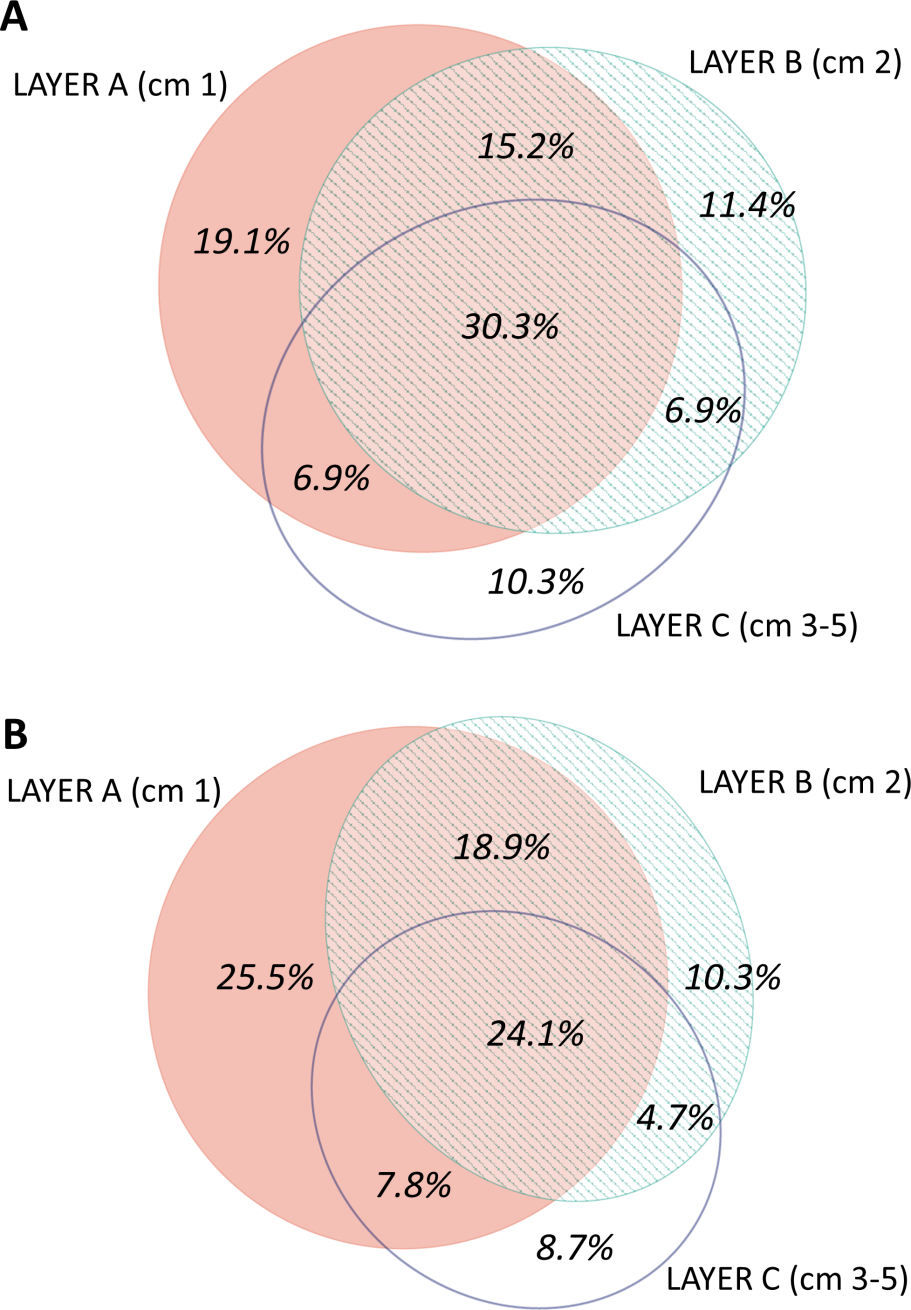
Venn diagram of the number of MOTUs found in the three layers of sediment in (A) the DNA and (B) the RNA datasets, all samples pooled. Areas drawn to scale.

The distribution by layer of taxa obtained from RNA and DNA (Fig. 8) showed that in both cases the number of MOTUs decreased from the surface to the deeper sediment, but this trend was more marked in the RNA dataset: the number of MOTUs in Layer C was 45.3% of the total, while for DNA it was 54.4%. Likewise, the number of exclusive MOTUs in layer C was 8.7% in the RNA template, and 10.3% with the DNA. On the other hand, the number of MOTUs shared by the three layers was lower (24.1%) in the RNA than in the DNA dataset (30.3%). When comparing the diversity of the different groups, both templates captured mostly the same composition for MOTUs that are found in only one layer (Fig. S7), while for MOTUs shared by the three layers there was a strong overrepresentation of most groups in the DNA dataset. A noticeable exception were the nematodes, which showed a higher number of MOTUs occurring in the three sediment layers with the RNA template (Fig. S7).

**Figure 9:**
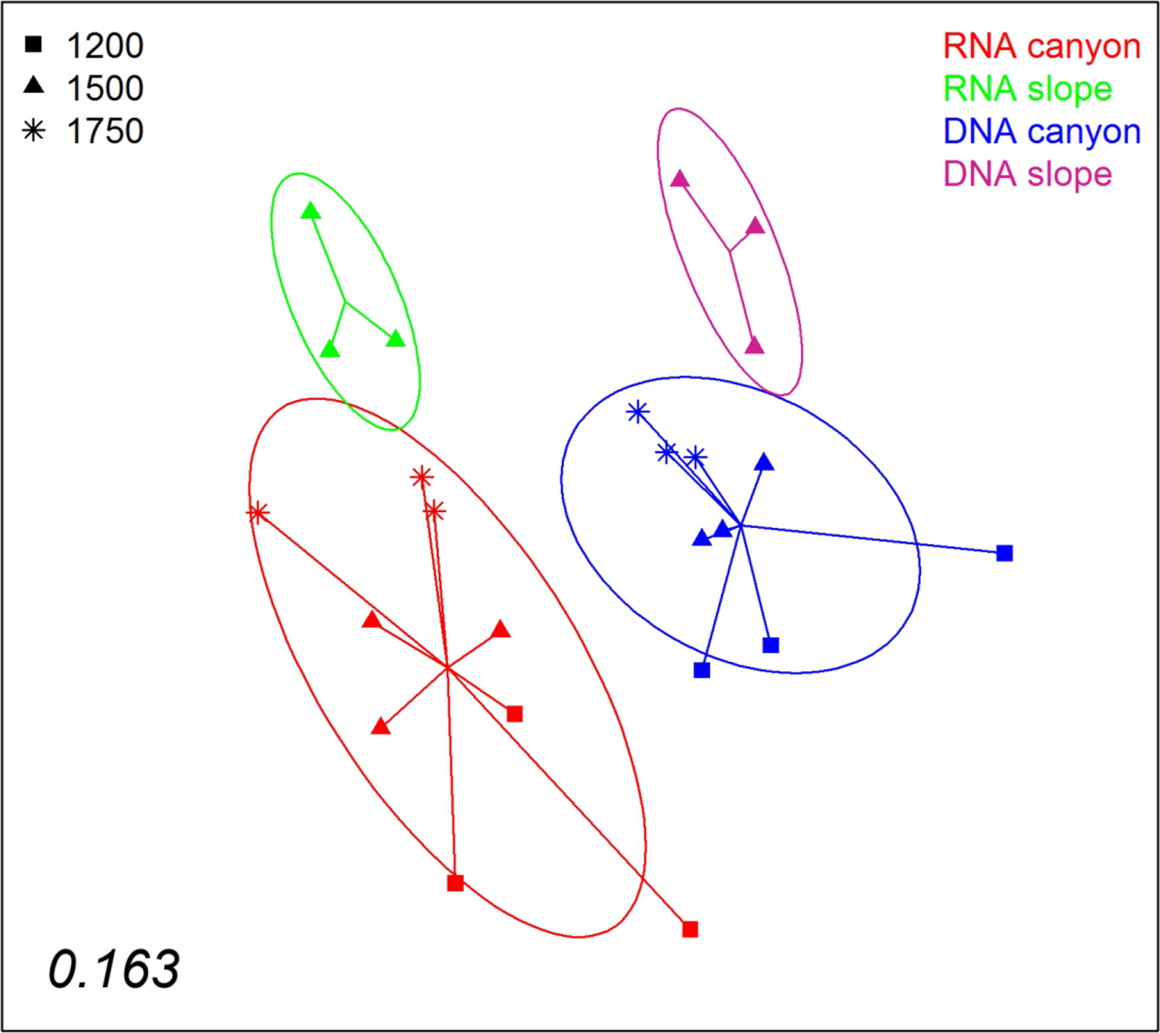
nMDS representation of the samples from which DNA and RNA was co-extracted (the three layers per sample pooled). The number in the lower left corner indicates the stress value of the final configuration.

A nMDS ordination of the samples using the Jaccard index (pooling layers) showed both datasets clearly differentiated (Fig. 9). Within each, the configurations were similar, showing separation between slope and canyon, and a depth gradient inside the canyon. However, the community structure captured with eDNA and eRNA was significantly different, with no overlap of the inertia ellipses. This result was confirmed in a PERMANOVA with type of nucleic acid (RNA or DNA) and zone as factors (Table S5). Both main factors were highly significant while the interaction was not, and no significant differences in dispersion values were detected.

## Discussion

The sheer diversity of sediment communities and the small size of many of the organisms involved represent important problems for traditional studies, and metabarcoding is a powerful tool for community profiling and analysis of distribution patterns in these communities (reviewed in Dafforn et al., 2014; Lallias et al., 2015; Carugati et al., 2015; Leray & Knowlton, 2016; Sinniger et al., 2016). However, caution is necessary when dealing with information derived from metabarcoding studies. The resolution of this approach depends on the marker used and the reference databases available for this marker. There is up to now a prevalent use of the 18S rRNA gene, but several regions are used in different studies, each coming with its particularities in terms of length, universality of the primers, degree of variability, and coverage of the databases. It has been established that 18S in general underestimates the true number of species (Tang et al., 2012; Leray & Knowlton, 2016). In our case, we have chosen a short hypervariable region (allowing amplification of even degraded DNA) with high *in silico* amplification success (Guardiola et al., 2015). On the downside of it, manual checking showed that in many groups different species can share the same sequence, thus our diversity estimates are on the conservative side. If the degree of underestimation is not biased among groups, then comparisons of taxonomic richness can still be carried out confidently. It is not possible at present to estimate such potential biases, but we used a flexible Bayesian method for delimiting MOTUs that should be able to accommodate differences between groups and minimize biases.

In addition, no metabarcoding study is better than the database it uses in terms of taxonomic assignment, and for many small groups there are large gaps in the databases, let alone for their deep sea representatives (Sinniger et al., 2016). Our taxonomic assignments (Figs. S3 and S4) should therefore be taken with caution, especially those at low levels. An approximate picture of the degree of completeness of the reference databases is given by the similarity of MOTUs to their respective best-matches in the reference database (Table S1). The mean similarities are always below the levels useful at low taxonomic ranges. Amebozoa, Excavata and Rhizaria have mean best matches below 0.9 similarity, indicating poor ability to adequately identify these taxa. Within metazoans, only Mollusca, Cnidaria, Echinodermata and Porifera have mean best matches above 0.97 (excluding Rotifera, with a mean of 0.995, but with only 2 MOTUs). Platyhelminthes (0.887), Xenacoelomorpha (0.911) and Nematoda (0.914) showed the lowest similarity values, indicating that the lineages found in our samples are poorly represented in the databases. However, one important advantage of metabarcoding is that, even if many MOTUs can be reliably assigned only at high taxonomic levels, they are given a genetic tag that makes them recognizable and usable in studies at other places or times. In addition, these MOTUs may be more precisely identified in the future, as reference databases grow denser.

Even with these caveats in mind, the results of this study support the use of our metabarcoding approach to monitor spatial and temporal variability in deep-sea sediment communities. We found a high diversity (over 5,500 MOTUs identified), being Metazoa, Alveolata, Rhizaria, and Stramenopiles the most diverse groups. Among Metazoa, the most diverse groups were Nematoda, Arthropoda, Annelida and Platyhelminthes, a result in agreement with previous metabarcoding studies of marine sediments (reviewed in Leray & Knowlton, 2016). We have also found an unexpected richness of Xenacoelomorpha (61 MOTUs, Table S1), a group whose hidden diversity starts to be uncovered by metabarcoding (Arroyo et al., 2016).

We found a high taxon turnover (*β*-diversity) in our samples, and significant layer, zone, depth, and time effects were detected, highlighting the complex dynamics of deep-sea communities. In a previous study (Guardiola et al., 2015), we analysed the sediment communities in several deep-sea canyons in the Northwestern Mediterranean Sea (the Blanes canyon among them) by targeting specifically the extracellular DNA contained in the sediments using a fast extraction protocol (Taberlet et al., 2012b). In the present paper, our extraction protocol with a lysis step captures total (both intra- and extracellular) DNA present in the sediment. Some samples in Guardiola et al. (2015) were taken at the same points inside the canyon sampled here but in spring 2012. Although taken at different years, comparison among the two methods in samples from the same localities and season can be revealing. Using Metazoa as an instance, we compared the proportion of MOTUs of the different Phyla in our samples of spring 2013 inside the canyon with samples from the same localities in Guardiola et al. (2015). The richness patterns showed striking differences in several Phyla, in particular a marked underrepresentation of nematodes and an overrepresentation of sponges in the extracellular DNA dataset (Fig. S8). Annelida, Arthropoda and Platyhelminthes were also significantly better represented in extracellular DNA. Sponges have been reported to expel continuously cellular components into the water (De Goeij et al., 2013) and can thus generate extracellular DNA at a fast rate. Nematodes are enclosed in proteinaceous cuticles that may act as barriers and reduce the amount of free DNA of nematode origin in the environment. Nematodes are the main group of metazoans in meiofaunal assemblages, where their abundances are of the order of 1–12 millions per square meter of sea floor (Giere, 2009). They reach densities of hundreds of individuals/cm^2^ in the sediment bottoms studied here (Romano et al., 2013). The underrepresentation of nematodes in extracellular DNA suggests that the extraction method used in the present work is more adequate for characterizing the sediment communities and we favour its use in future research.

A strong degree of heterogeneity in community structure was found at all scales examined. The *β*-diversity estimates (Fig. 6) showed that, on average, only 32% of MOTUs were shared between samples from the same locality. The sequencing depth per sample was adequate, reaching MOTU saturation in most cases, so the low similarities found are likely related to the marked heterogeneity of sediment communities. It seems advisable to increase the number of replicates per locality in future studies to better capture the biodiversity present.

The vertical structure of the sediment was reflected in decreasing taxonomic richness from the surface down to 5 cm, with the three layers analysed harbouring significantly different communities. Our finding is consistent with the known decreasing abundance of meiofauna in the first centimetres of sediment (e.g., Kalogeropoulou et al., 2016; Romano et al., 2013). The analysis of RNA, which should correspond to the live fraction, showed less biodiversity at the deeper sediment layer than the analysis of DNA, suggesting some degree of transport of DNA from dead organisms from upper strata, similar to the “DNA leaching” reported in terrestrial soils (e.g., Andersen et al., 2012). The RNA recovered in general less MOTUs shared by the three layers of sediment, indicating that many shared MOTUs found with DNA do not actually live in the three sediment layers. Only for Nematoda did the richness of shared MOTUs recovered with RNA exceed that of DNA, suggesting that this group is particularly capable of active vertical movements in the sediment, as has been noted in shallower sediments (e.g., Steyaert et al., 2001; Brustolin, Thomas & Lana, 2013). The use of metabarcoding for assessing vertical distribution and tracing movements of organisms in the sediment as a response to environmental changes (e.g., Lambshead, Ferrero & Wollf, 1995) is a promising field that deserves further exploration.

The extraction procedure used allowed us to obtain eDNA and eRNA from exactly the same sediment sample. The former includes DNA from living organisms, as well as DNA resulting from organisms’ remains, exudates, or free DNA. Sediment DNA can be preserved adsorbed to particles from periods that vary over orders of magnitude as a function of environmental parameters, but that can reach thousands of years, particularly in anoxic sediments (Corinaldesi, Barucca & Dell’Anno, 2011; Thomsen & Willerslev, 2015; Barnes & Turner, 2016). Even in the well-oxygenated top centimetre of marine sediments, residence times for DNA reach ca. 10 years (Dell’Anno & Danovaro, 2005). Our DNA template, therefore, comprises both DNA from present and past organisms inhabiting the sediment and DNA “snowfall” from planktonic organisms. The RNA template, on the other hand, reflects preferentially the benthic organisms alive and active at the time of collection.

As expected, with a similar number of reads, we found 20.8% more total MOTUs in the DNA than in the RNA dataset. We also found 41.9 % more MOTUs per sample with the DNA template, indicating that the DNA was more spread among samples, as can be expected if it included extra-organismal and extracellular components. More MOTUs had been found with DNA for most taxa, but RNA uncovered a higher richness of Amoebozoa and Nematoda than the DNA samples. The MOTUs found only in RNA were in general rare MOTUs with few reads, which may explain why they had not been amplified in the more heterogeneous DNA dataset. Our nMDS analyses indicated that RNA-derived taxa showed a similar spatial configuration with respect to depth and zone than the DNA-derived taxa. However, both datasets appeared well separated in nMDS ordinations, indicating different community composition. In fact, only 55.9% of the MOTUs were shared by both datasets.

Previous studies reported overall similar communities of coastal marine protists recovered from DNA and RNA templates, with some revealing exceptions in particular groups (Logares et al., 2014; Massana et al., 2015; Lejzerowicz, Volstky & Pawlowski, 2013). Our results showed that eDNA and eRNA capture different community composition, affording useful information in particular for groups showing discordances for both templates. They should be considered complementary tools and used in parallel whenever possible. Both showed, however, similar patterns of ecological differentiation with season and depth, and there was no evidence that spatial patterns were blurred by “dead” DNA persistence and transport. Thus, DNA may be favoured for routine studies given that sample preservation is more complex with RNA, especially if using liquid nitrogen. In addition, current commercial kits allow extraction of RNA from only small amounts of sediment. It must also be noted that ribosomal RNA may be more stable than previously thought, particularly in sediments (Orsi, Biddle & Edgcomb, 2013). Further comparative studies of the stability of ribosomal and messenger RNA under different conditions are necessary to establish the best proxy for living, active biomass.

Metabarcoding has been rarely used to assess temporal changes in marine eukaryotic communities (e.g., Brannock et al., 2014; Massana et al., 2015; Brannock et al., 2016; Chain et al., 2016). Our results showed that samples from the same localities at different seasons were well differentiated, both in the canyon and in the adjacent slope. Unfortunately, having sampled only one season each, we cannot assign the differences found to seasonal patterns, but they reveal a strong temporal component of variation in these communities. We also found that, in the canyon, the autumn communities sampled were more heterogeneous than in spring. In terms of Super-Groups, we detected significantly more MOTUs of Alveolata and Metazoa in spring and, among the latter, the main effect was a marked increase in nematode diversity and abundance. This is in agreement with previous studies reporting higher meiofaunal abundances in the canyon axis in spring than in autumn (Romano et al., 2013). In general, meiofaunal biomass is related to food inputs provided by pulses of settling organic matter, to which benthic communities respond with some time lag (Gooday, 2002). In the Northwestern Mediterranean Sea, there are strong seasonal signatures in the input of particles to the sediment bottoms (Guidi-Guilvard et al., 2009), with marked peaks in winter and spring. In addition, episodic downward fluxes linked to river discharges, storms and cascading events occur also in winter in the Blanes Canyon (Zúñiga et al., 2009), thereby transporting large amounts of organic matter to the deep. This increased availability of food may have a homogenizing effect, while in autumn, under less favourable trophic conditions, the communities become more heterogeneous.

We found significant differences in community structure between the open slope and comparable depths inside the canyon at the two time points analysed. Depth appeared in our analyses as a major factor determining community composition within the canyon, with significant differences when comparing samples separated by more than one depth level. Several biogeochemical variables may co-vary with depth and be responsible for this pattern, although previous studies in the Blanes canyon showed relatively homogeneous parameters within the range of depths here analysed. For instance, the sediment showed a slight increase in the clay fraction (35–41% from the entrance of the canyon to the deep zones), with a concomitant decrease in silt (63–59%) (Ingels et al., 2013). Total organic carbon ranged from 0.75 to 0.81% inside the canyon with no clear bathymetric trend, while total nitrogen was 0.09% at all depths (Ingels et al., 2013; Romano et al., 2013). Community changes may be more related to sediment fluxes, which increased with depth along the canyon axis (mass fluxes from 12.68 g m^−2^ d^−1^ to 26.57 g m^2^d^−1^), while the organic contents of these fluxes likewise increased with depth (from 1.68 to 2.21%) (Lopez-Fernandez et al., 2013). Bathymetric patterns of biodiversity in the deep Mediterranean Sea are variable, but significant changes in species composition with depth are the norm (Danovaro et al., 2010). The interplay of episodic pulses of detrital input and disturbances such as water cascading, acting on a topographically heterogeneous environment, can explain the spatio-temporal trends of communities in the canyon. The information obtained from genetic data, if gathered over time and coupled with physico-chemical parameters, can contribute to our understanding of the dynamics of these deep-sea communities.

In conclusion, with our metabarcoding approach we could detect a high degree of biodiversity, at over 5,500 MOTUs on a single canyon. Findings of thousands of MOTUs are common in metabarcoding studies of sediments (e.g., Fonseca et al., 2014; Lallias et al., 2015; Chariton et al., 2015; Sinniger et al., 2016). Our data unravelled heterogeneity at several scales (spatial and temporal) in deep-sea canyon communities, considered a hot-spot of biodiversity and, in the area studied, subject to anthropogenic threats (e.g., Palanques et al., 2006; Puig et al., 2012; Pusceddu et al., 2014). We postulate that continued monitoring of these communities using metabarcoding will provide basic information for taking appropriate management actions. As databases get denser and molecular tags can be linked to morphologically identified individuals this information will become even more valuable.

Our results indicate that the use of extraction procedures with a lysis step is advisable, that emphasis should be placed on replication within localities, and that eRNA and eDNA, whenever possible, should be studied in parallel. We have used a novel marker fragment and an improved pipeline that may be applicable in future studies in this and other areas. Environmental DNA has already produced important insights applicable to management and conservation (reviewed in Thomsen & Willerslev, 2015; Handley, 2015), and eDNA monitoring is already being adopted for ecological surveys (e.g., in the UK, Rees et al., 2014). A continuously improved metabarcoding tool will undoubtedly become a cornerstone in deep-sea biomonitoring and management.

##  Supplemental Information

10.7717/peerj.2807/supp-1 Figure S1 Graphical representation of the “ cleaning by similarity ” step performed It can be seen how the sequences that lacked a match in the database were mainly long sequences, pruned at this step. Note different scales in *Y*-axis.

10.7717/peerj.2807/supp-2 Figure S2 Rarefaction curves of the number of MOTUs obtained at increasing numbers of reads in the samples The vertical line marks the mean number of reads obtained per sample.

10.7717/peerj.2807/supp-3 Figure S3 Proportion of MOTUs assigned at the different taxonomic levels

10.7717/peerj.2807/supp-4 Figure S4 Number of taxa belonging to the different taxonomic categories that were identified for the whole dataset of Metazoa.

10.7717/peerj.2807/supp-5 Figure S5 Venn diagram of the number of MOTUs in the three layers of sediment sampled All samples pooled. Areas drawn to scale.

10.7717/peerj.2807/supp-6 Figure S6 Number of taxa of the different taxonomic categories identified in the samples for which RNA and DNA was extracted (A) Metazoa ; (B) Nematoda.

10.7717/peerj.2807/supp-7 Figure S7 Number of MOTUs in the RNA and DNA datasets Comparisons of the number of MOTUs of the different Super-Groups (A) and metazoan Phyla (B) that occur on a single layer of sediment (“Non- shared ”) or at the three layers (“ Shared ”).

10.7717/peerj.2807/supp-8 Figure S8 Proportionof MOTUs of the metazoan Phyla found with ourextraction method including a lysis step (“total” DNA) and that of Guardiola et al. (2015) (“extracellular” DNA) S amples were obtained at the same localities in the Blanes Canyon in spring 2013 and 2012, respectively. Bi-directional bars are standard errors. Taxa showing significant differences among methods (*t*-tests) are colour –coded and listed in legends.

10.7717/peerj.2807/supp-9 Table S1 Number of MOTUs and reads found for the Supergroups considered Data are further subdivided by Phyla for the Metazoa. RNA and DNA datasets refer to the samples for which both nucleic acids had been co-extracted. Total columns refer to all datasets pooled. Unassigned means metazoan MOTUs (reads) that could not be reliably assigned to any given Phylum by the ecotag procedure. Mean best hit is the mean value of similarity of MOTUs to their best matches in the reference database.

10.7717/peerj.2807/supp-10 Table S2 PERMANOVA and PERMDISP tests of the factor Layer ( with Locality as nested factor) for the Jaccard index The results for permutational pairwise tests of levels of the factor Layer are also provided (*: significant outcome after FDR correction).

10.7717/peerj.2807/supp-11 Table S3 PERMANOVA and PERMDISP tests of the effect of Zone (canyon and slope) and Season (autumn and spring) for the Jaccard index Only canyon samples from 1,500 m depth were included in the analyses. The three layers of each sample pooled.

10.7717/peerj.2807/supp-12 Table S4 PERMANOVA and PERMDISP tests of the effects of Season and Depth on the samples from the Blanes Canyon for the Jaccard index The results for permutational pairwise tests of levels of the factor Depth are also provided (*: significant outcome after FDR correction). The three layers of each sample pooled.

10.7717/peerj.2807/supp-13 Table S5 PERMANOVA and PERMDISP tests of the effects of the type of nucleic acid (RNA or DNA) and Zone (canyon, slope) for the Jaccard index The three layers of each sample pooled.

## Additional Information and Declarations

### DNA Deposition

The following information was supplied regarding the deposition of DNA sequences: Dryad doi: 10.5061/dryad.4kn05.

### Data Availability

The following information was supplied regarding data availability: The raw data is located at Dryad doi: 10.5061/dryad.4kn05.
