# Supplementary figures and images for: Spatio-temporal monitoring of deep-sea communities using metabarcoding of sediment DNA and RNA
Source: PeerJ. 2016 Dec 21;4:e2807. doi: 10.7717/peerj.2807 (PMC5180584; doi:10.7717/peerj.2807)

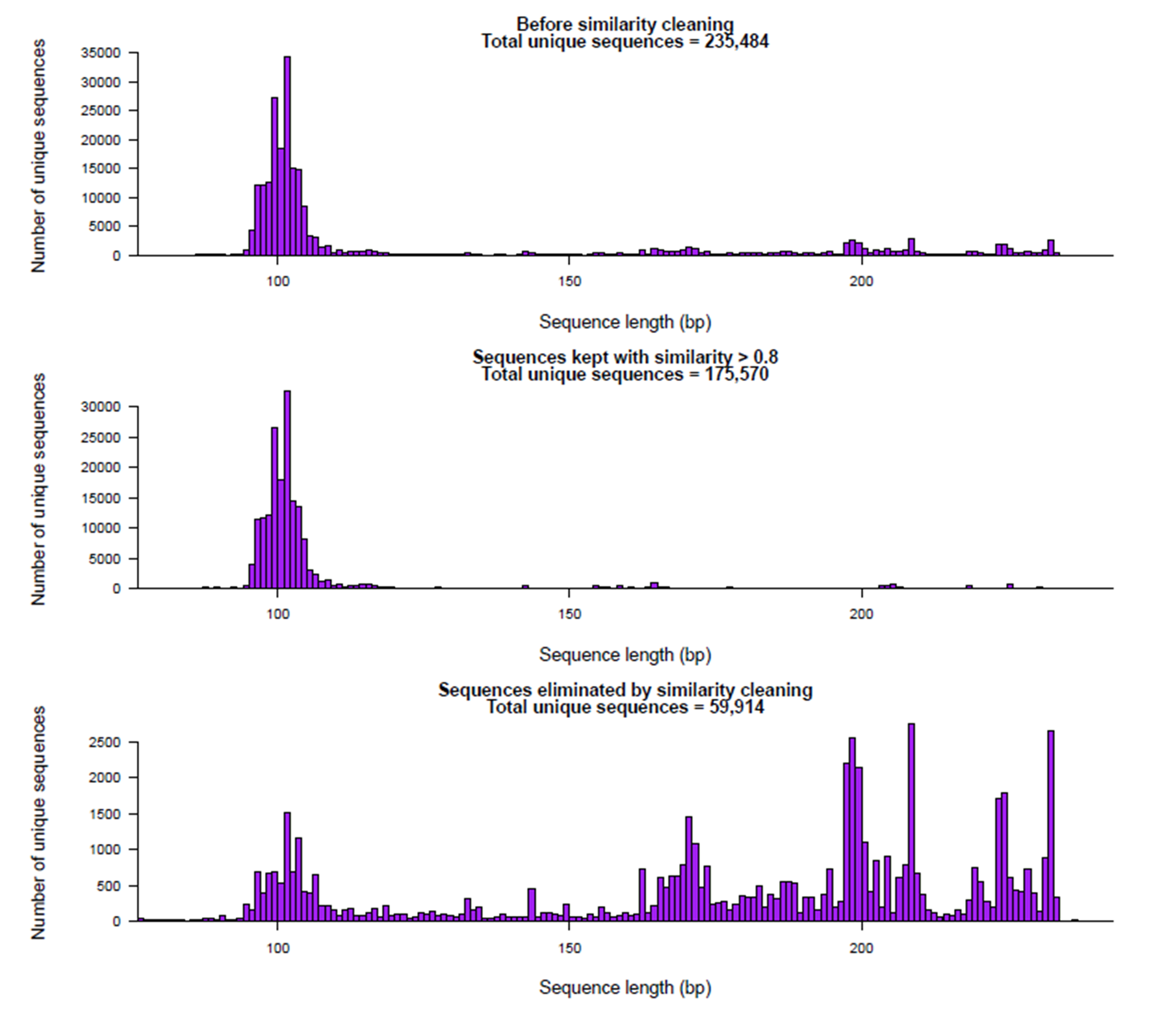

Supplement: Figure S1 — It can be seen how the sequences that lacked a match in the database were mainly long sequences, pruned at this step. Note different scales in Y-axis. [file peerj-04-2807-s001.png]

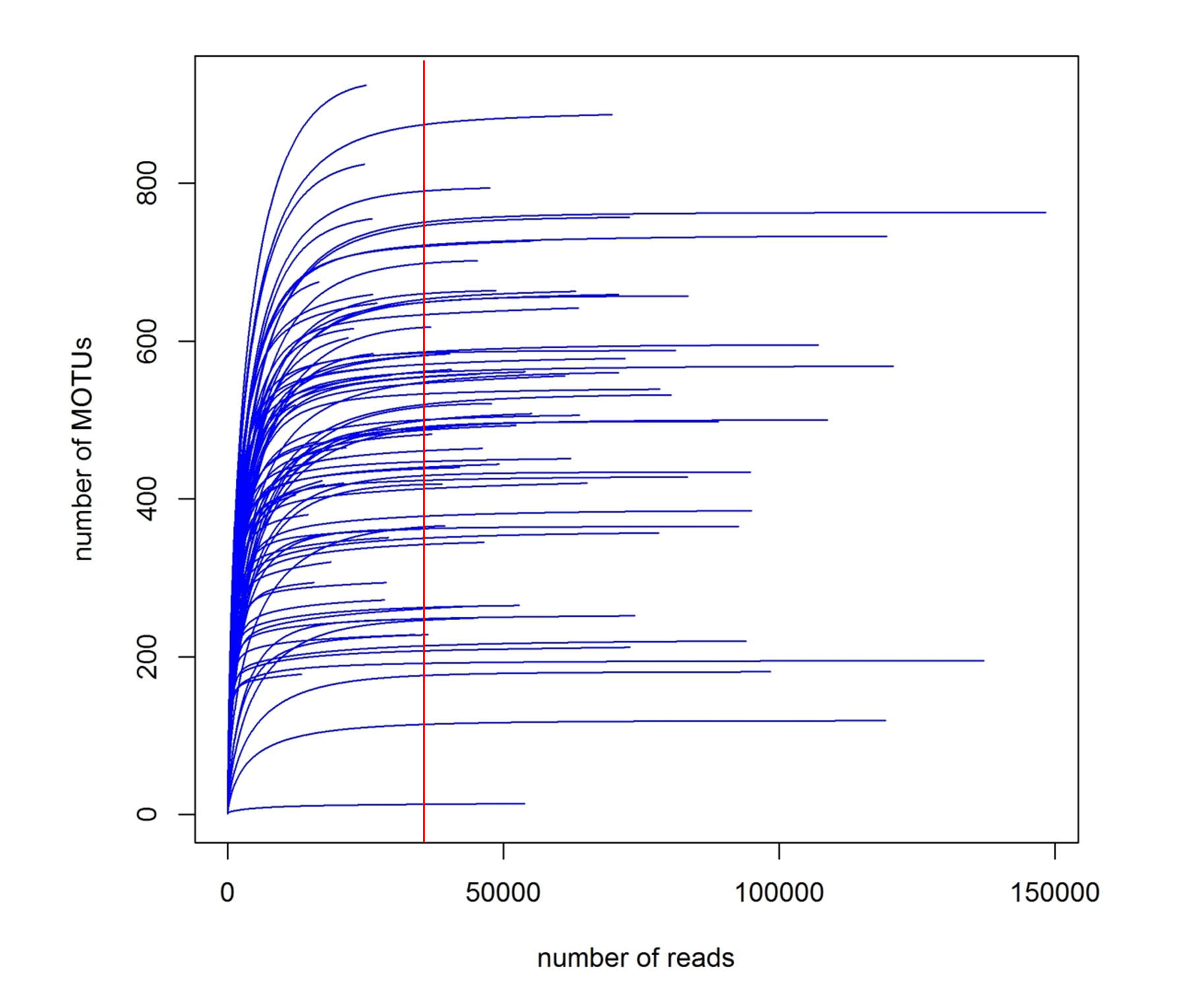

Supplement: Figure S2 — The vertical line marks the mean number of reads obtained per sample. [file peerj-04-2807-s002.png]

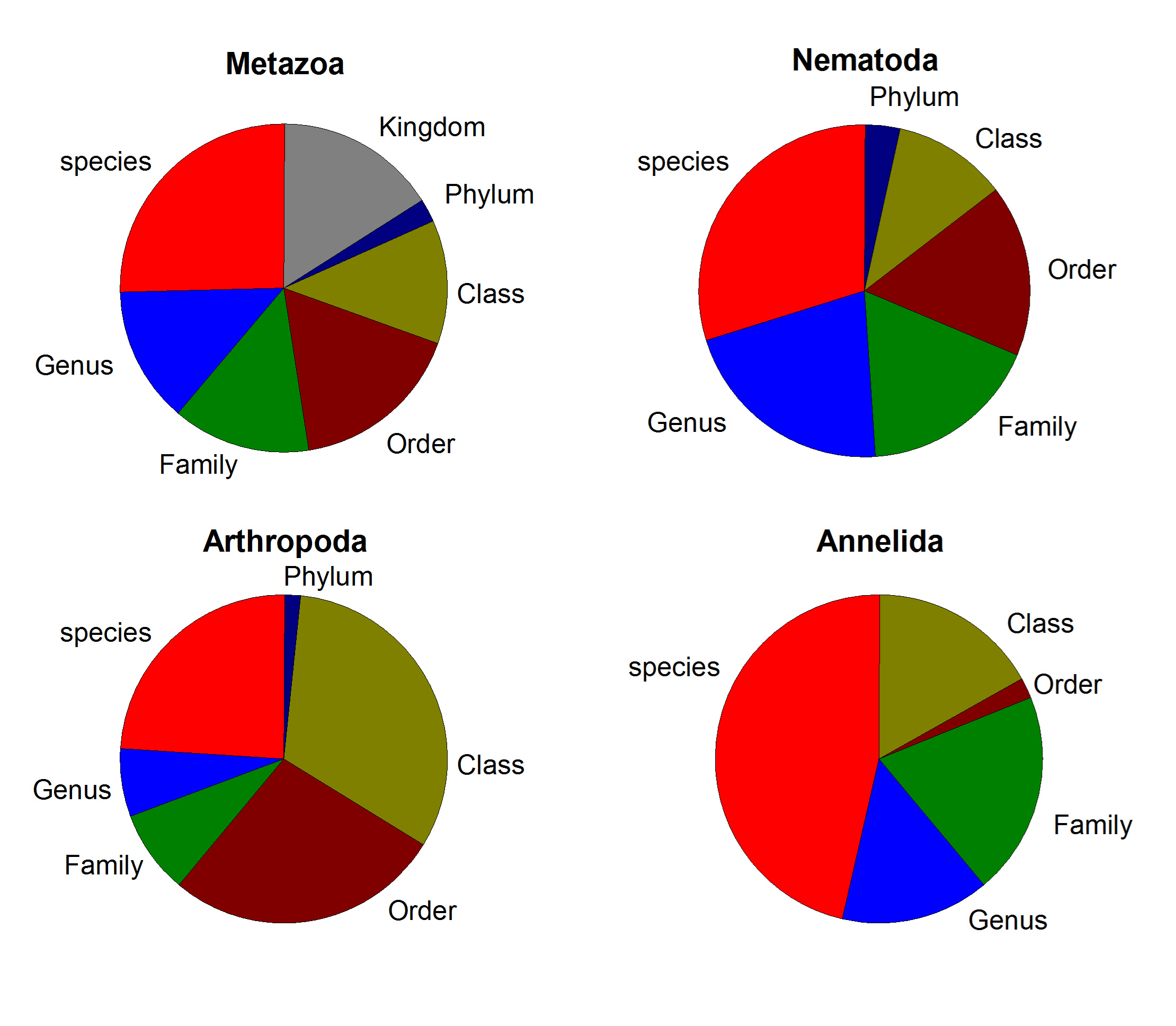

Supplement: Figure S3 [file peerj-04-2807-s003.png]

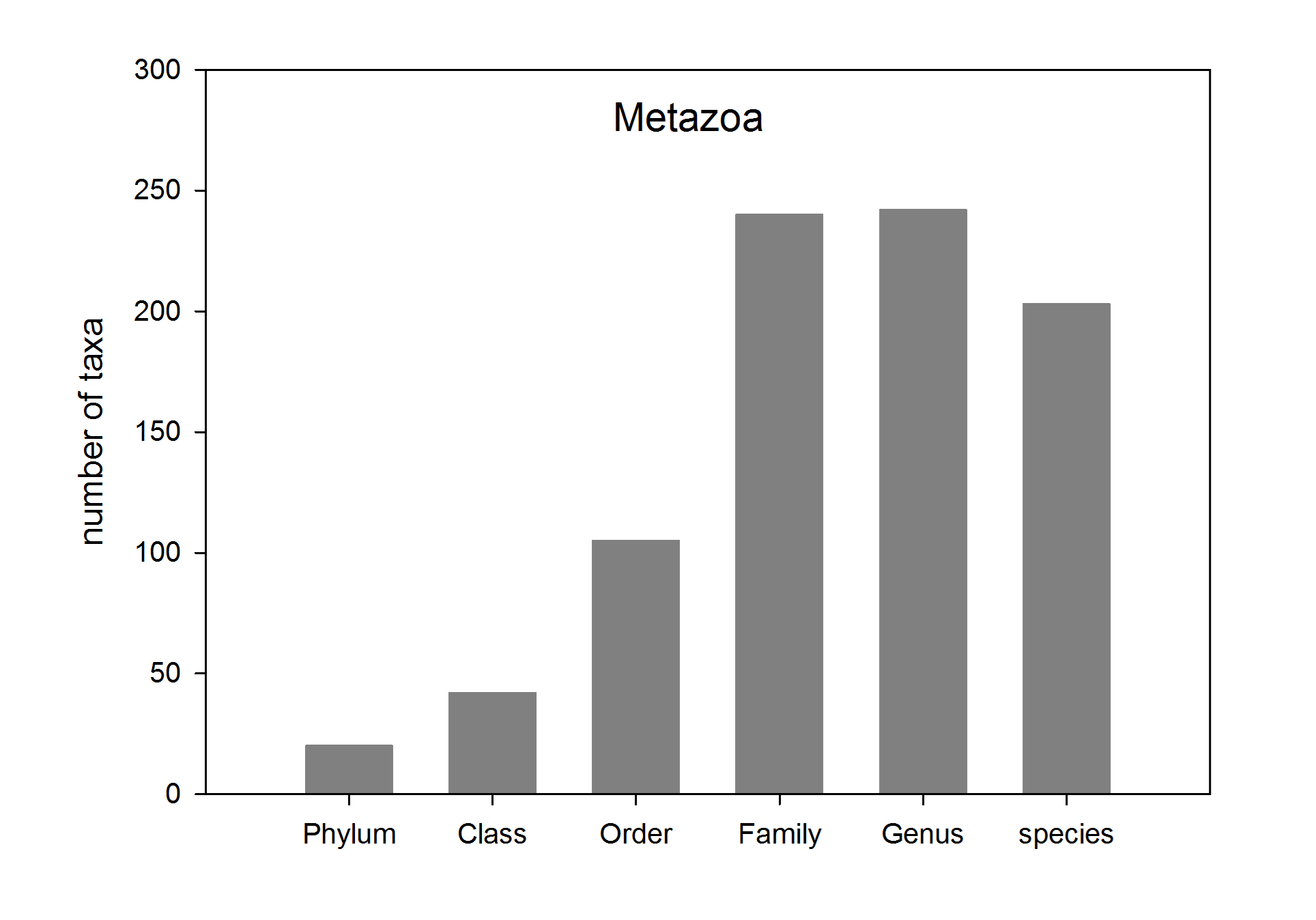

Supplement: Figure S4 [file peerj-04-2807-s004.png]

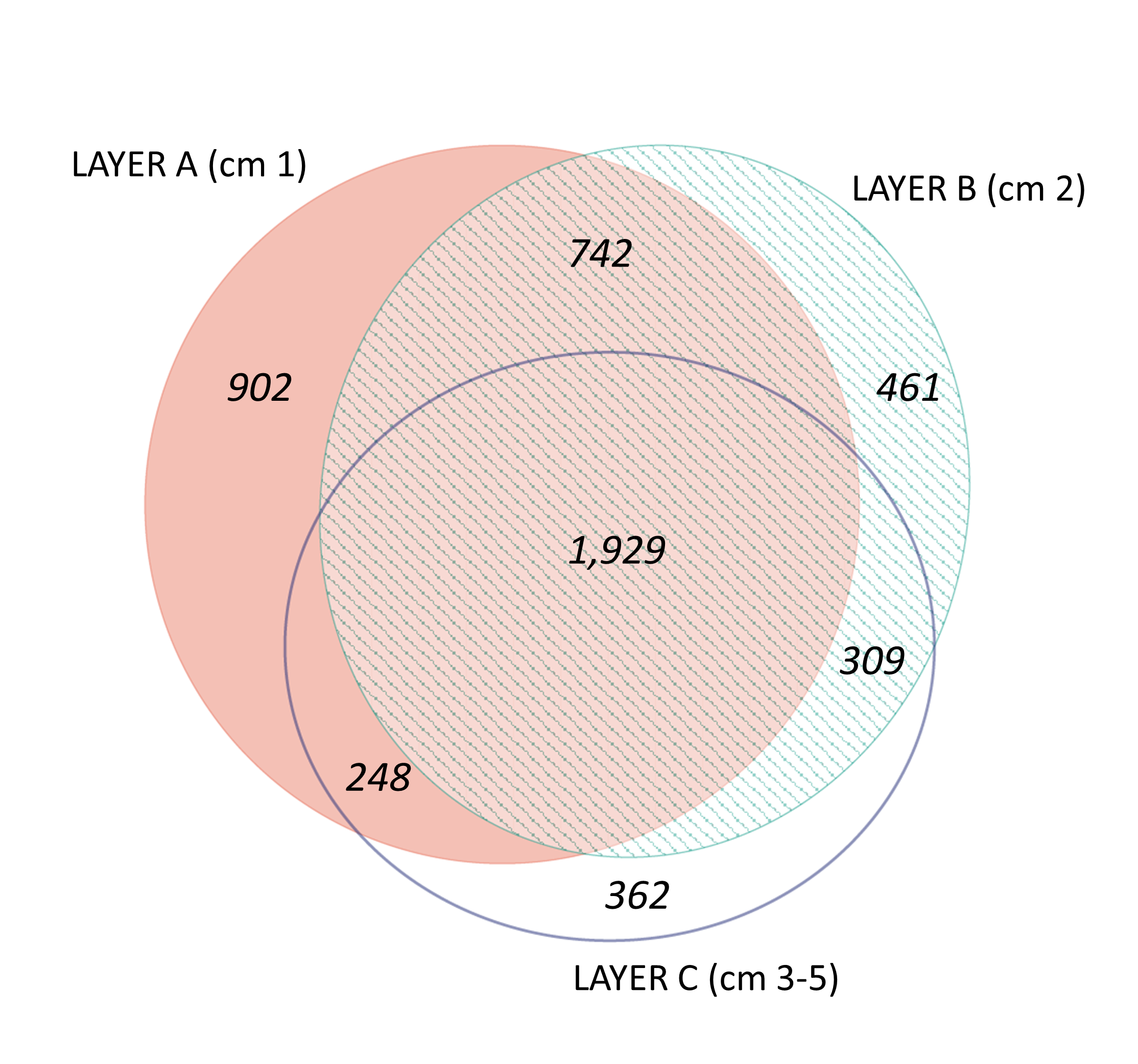

Supplement: Figure S5 — All samples pooled. Areas drawn to scale. [file peerj-04-2807-s005.png]

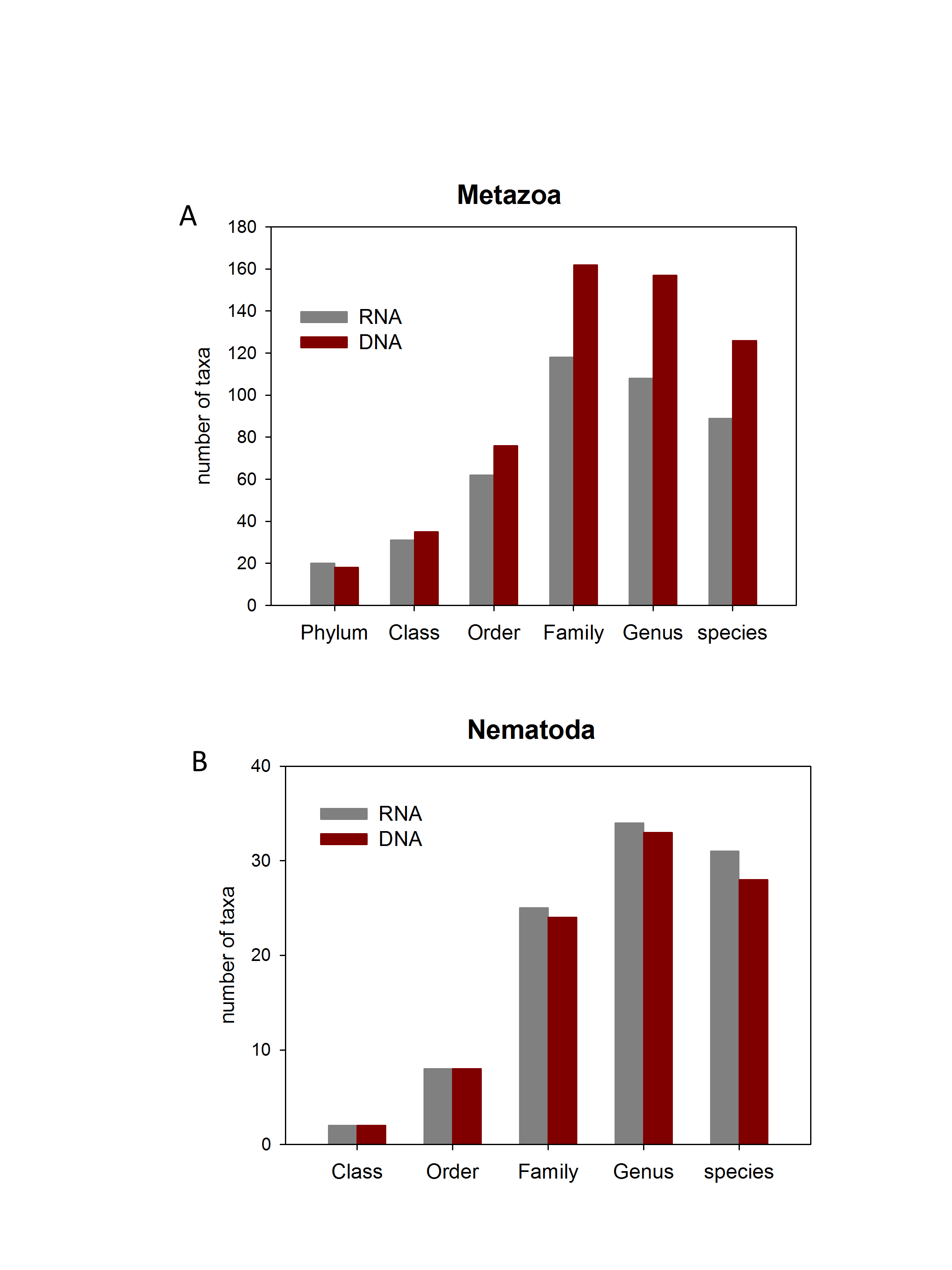

Supplement: Figure S6 — (A) Metazoa ; (B) Nematoda. [file peerj-04-2807-s006.png]

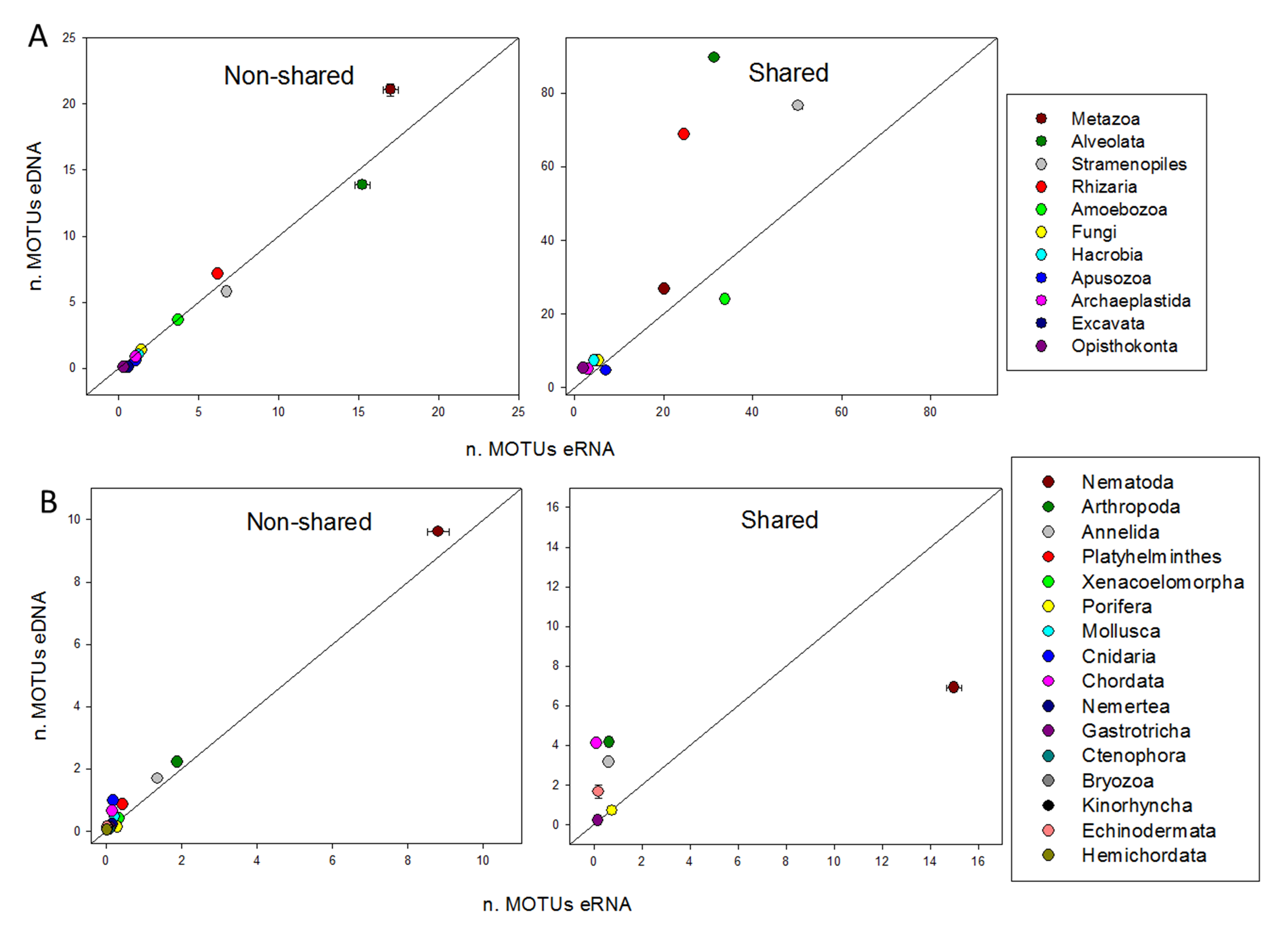

Supplement: Figure S7 — Comparisons of the number of MOTUs of the different Super-Groups (A) and metazoan Phyla (B) that occur on a single layer of sediment (“Non- shared ”) or at the three layers (“ Shared ”). [file peerj-04-2807-s007.png]

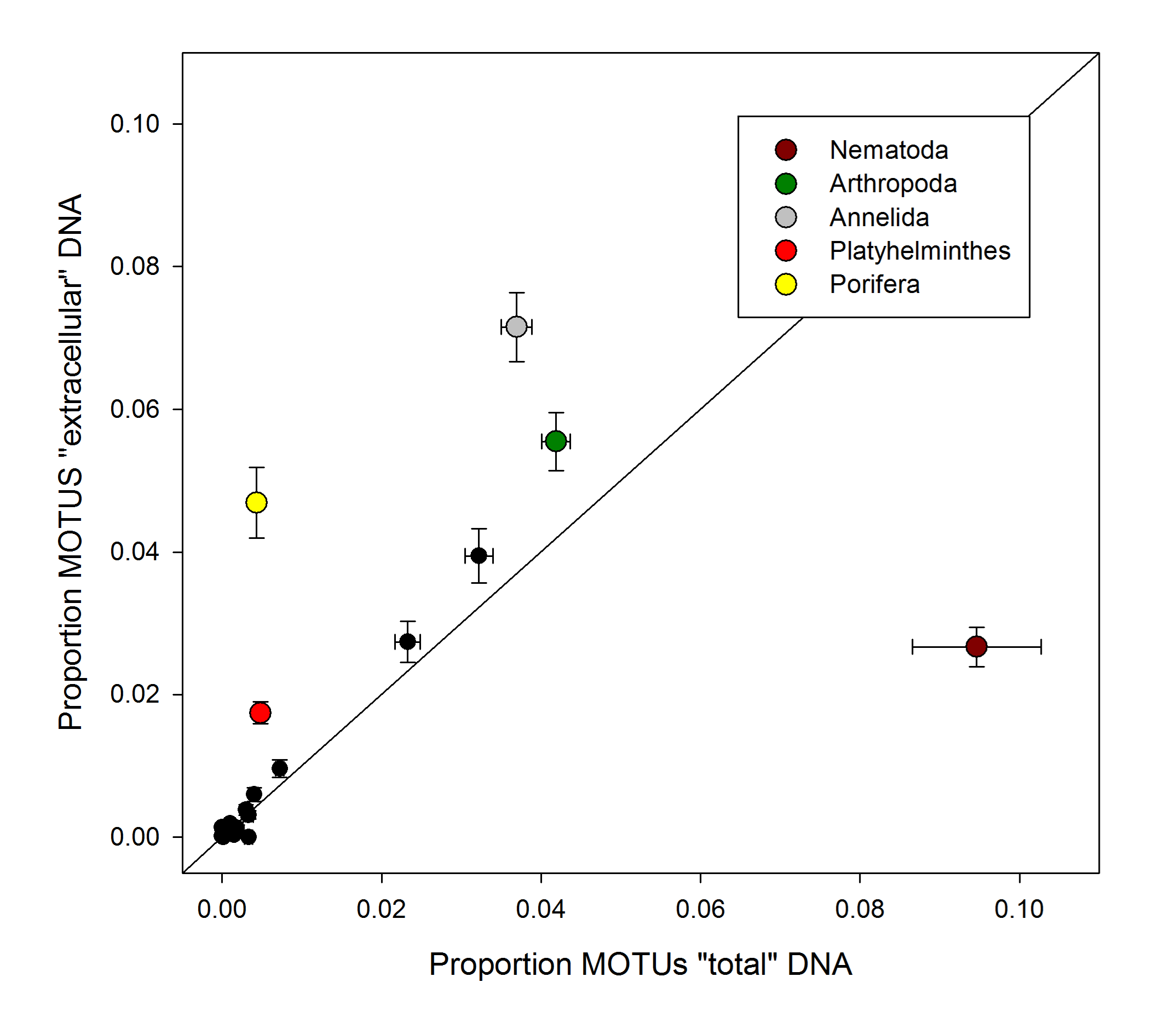

Supplement: Figure S8 — S amples were obtained at the same localities in the Blanes Canyon in spring 2013 and 2012, respectively. Bi-directional bars are standard errors. Taxa showing significant differences among methods (t-tests) are colour –coded and listed in legends. [file peerj-04-2807-s008.png]
