# Supplementary material for: Spatio-temporal monitoring of deep-sea communities using metabarcoding of sediment DNA and RNA
Source: PeerJ. 2016 Dec 21;4:e2807. doi: 10.7717/peerj.2807 (PMC5180584; doi:10.7717/peerj.2807)
Supplement: Table S1 — Data are further subdivided by Phyla for the Metazoa. RNA and DNA datasets refer to the samples for which both nucleic acids had been co-extracted. Total columns refer to all datasets pooled. Unassigned means metazoan MOTUs (reads) that could not be reliably assigned to any given Phylum by the ecotag procedure. Mean best hit is the mean value of similarity of MOTUs to their best matches in the reference database. [file peerj-04-2807-s009.docx]

| SUPERGROUPS | MOTUs Autumn | reads Autumn | MOTUs Spring | reads Spring | MOTUs RNA | reads RNA | MOTUs DNA | reads DNA | Total MOTUs | Total reads | mean best hit |
| --- | --- | --- | --- | --- | --- | --- | --- | --- | --- | --- | --- |
| Alveolata | 1,117 | 439,284 | 1,160 | 274,988 | 685 | 51,953 | 985 | 96,335 | 1,480 | 862,560 | 0.907 |
| Amoebozoa | 200 | 39,408 | 211 | 16,891 | 288 | 18,821 | 229 | 9,061 | 328 | 84,181 | 0.860 |
| Apusozoa | 46 | 5,712 | 59 | 4,380 | 52 | 2,760 | 55 | 1,414 | 64 | 14,266 | 0.934 |
| Archaeplastida | 67 | 8,853 | 76 | 5,788 | 58 | 3,051 | 66 | 1,892 | 98 | 19,584 | 0.916 |
| Choanoflagellate | 16 | 12,694 | 17 | 8,802 | 16 | 613 | 17 | 3,520 | 17 | 25,629 | 0.969 |
| Excavata | 23 | 236 | 21 | 359 | 25 | 101 | 6 | 19 | 43 | 715 | 0.878 |
| Fungi | 75 | 23,052 | 74 | 10,266 | 97 | 51,925 | 78 | 17,875 | 136 | 103,118 | 0.943 |
| Hacrobia | 79 | 15,851 | 85 | 7,384 | 63 | 3,176 | 67 | 2,481 | 101 | 28,892 | 0.923 |
| Metazoa | 1,230 | 1,642,963 | 1,327 | 1,039,932 | 878 | 304,870 | 996 | 253,575 | 1,881 | 3,241,340 | 0.918 |
| Rhizaria | 613 | 571,074 | 596 | 229,070 | 327 | 20,649 | 550 | 69,757 | 740 | 890,550 | 0.896 |
| Stramenopiles | 502 | 173,274 | 499 | 155,196 | 442 | 60,986 | 493 | 68,510 | 681 | 457,966 | 0.910 |
| TOTAL | 3,968 | 2,932,401 | 4,125 | 1,753,056 | 2,931 | 518,905 | 3,542 | 524,439 | 5,569 | 5,728,801 |  |
| METAZOAN PHYLA |  |  |  |  |  |  |  |  |  |  |  |
| Annelida | 118 | 698,975 | 99 | 331,889 | 64 | 125,169 | 82 | 104,942 | 149 | 1,260,975 | 0.967 |
| Arthropoda | 164 | 156,239 | 163 | 153,486 | 86 | 16,763 | 118 | 31,799 | 255 | 358,287 | 0.933 |
| Bryozoa | 6 | 2,736 | 9 | 3,201 | 4 | 988 | 3 | 22 | 12 | 6,947 | 0.947 |
| Chaetognatha | 2 | 7 | 0 | 0 | 1 | 19 | 0 | 0 | 2 | 26 | 0.991 |
| Chordata | 54 | 64,251 | 49 | 26,572 | 12 | 280 | 41 | 2,278 | 59 | 93,381 | 0.967 |
| Cnidaria | 61 | 109,678 | 50 | 29,691 | 12 | 530 | 42 | 4,719 | 65 | 144,618 | 0.970 |
| Ctenophora | 7 | 1,530 | 6 | 504 | 4 | 42 | 6 | 161 | 7 | 2,237 | 0.957 |
| Echinodermata | 7 | 1,819 | 10 | 768 | 3 | 351 | 8 | 1,187 | 12 | 4,125 | 0.981 |
| Gastrotricha | 6 | 7,422 | 7 | 3,248 | 7 | 508 | 7 | 547 | 9 | 11,725 | 0.930 |
| Hemichordata | 6 | 8,597 | 6 | 12,606 | 3 | 173 | 4 | 527 | 7 | 21,903 | 0.949 |
| Kinorhyncha | 5 | 5,896 | 4 | 22,470 | 5 | 2,184 | 8 | 4,454 | 9 | 35,004 | 0.959 |
| Loricifera | 1 | 21 | 1 | 376 | 1 | 3 | 0 | 0 | 3 | 400 | 0.905 |
| Mollusca | 27 | 14,381 | 24 | 18,162 | 8 | 2,241 | 17 | 6,540 | 41 | 41,324 | 0.973 |
| Nematoda | 469 | 173,427 | 577 | 218,595 | 493 | 112,380 | 457 | 42,797 | 747 | 547,199 | 0.914 |
| Nemertea | 15 | 55,830 | 12 | 20,356 | 5 | 2,474 | 10 | 9,991 | 20 | 88,651 | 0.952 |
| Platyhelminthes | 68 | 70,594 | 63 | 57,089 | 21 | 12,595 | 37 | 14,864 | 123 | 155,142 | 0.887 |
| Porifera | 10 | 2,594 | 9 | 6,908 | 13 | 2,995 | 8 | 4,278 | 17 | 16,775 | 0.971 |
| Priapulida | 1 | 706 | 1 | 318 | 1 | 155 | 1 | 97 | 1 | 1,276 | 0.910 |
| Rotifera | 1 | 40 | 2 | 18 | 1 | 264 | 0 | 0 | 2 | 322 | 0.995 |
| Xenacoelomorpha | 30 | 17,129 | 39 | 10,852 | 11 | 1,963 | 16 | 3,373 | 61 | 33,317 | 0.911 |
| Unassigned | 172 | 251,091 | 196 | 122,823 | 123 | 22,793 | 131 | 20,999 | 280 | 417,706 | 0.856 |

Table S1. Number of MOTUs and reads found for the Supergroups considered. Data are further subdivided by Phyla for the Metazoa. RNA and DNA datasets refer to the samples for which both nucleic acids had been co-extracted. Total columns refer to all datasets pooled. Unassigned means metazoan MOTUs (reads) that could not be reliably assigned to any given Phylum by the ecotag procedure. Mean best hit is the mean value of similarity of MOTUs to their best matches in the reference database.
