# Supplementary material for: Spatio-temporal monitoring of deep-sea communities using metabarcoding of sediment DNA and RNA
Source: PeerJ. 2016 Dec 21;4:e2807. doi: 10.7717/peerj.2807 (PMC5180584; doi:10.7717/peerj.2807)
Supplement: Table S2 — The results for permutational pairwise tests of levels of the factor Layer are also provided (*: significant outcome after FDR correction). [file peerj-04-2807-s010.docx]

|  | *df* | *SS* | *Pseudo-F* | *P-value* | *Permdisp* |
| --- | --- | --- | --- | --- | --- |
| Layer | 2 | 16,145 | 2.362 | <0.001 | 0.104 |
| Locality | 9 | 46,235 | 1.499 | <0.001 | 0.588 |
| Corer (Locality) | 20 | 68,579 | 1.134 | <0.001 | 0.670 |
| Layer*Locality | 18 | 61,559 | 1.131 | 0.003 |  |
| Residual | 39 | 117,920 |  |  |  |
|  |  |  |  |  |  |
|  |  |  |  |  |  |
| *Layer Comparison* | *t* | *P-value* |  |  |  |
| A - B | 1.525 | 0.022* |  |  |  |
| A - C | 1.962 | <0.001* |  |  |  |
| B - C | 1.277 | 0.076 |  |  |  |
|  |  |  |  |  |  |

Table S2. PERMANOVA and PERMDISP tests of the factors Layer and Locality (with Corer as nested factor) for the Jaccard index. The results for permutational pairwise tests of levels of the factor Layer are also provided (*: significant outcome after FDR correction).
