# Supplementary material for: Spatio-temporal monitoring of deep-sea communities using metabarcoding of sediment DNA and RNA
Source: PeerJ. 2016 Dec 21;4:e2807. doi: 10.7717/peerj.2807 (PMC5180584; doi:10.7717/peerj.2807)
Supplement: Table S3 — Only canyon samples from 1,500 m depth were included in the analyses. The three layers of each sample pooled. [file peerj-04-2807-s011.docx]

|  | *df* | *SS* | *Pseudo-F* | *P-value* | *Permdisp* |
| --- | --- | --- | --- | --- | --- |
| Zone | 1 | 5,016 | 2.339 | 0.003 | 0.543 |
| Season | 1 | 2,980 | 1.389 | 0.036 | 0.007 |
| Zone*Season | 1 | 2,570 | 1.198 | 0.185 |  |
| Residual | 8 | 17,160 |  |  |  |

Table S3. PERMANOVA and PERMDISP tests of the effect of Zone (canyon and slope) and Season (autumn and spring) for the Jaccard index. Only canyon samples from 1500 m depth were included in the analyses. The three layers of each sample pooled.
