# Supplementary material for: Spatio-temporal monitoring of deep-sea communities using metabarcoding of sediment DNA and RNA
Source: PeerJ. 2016 Dec 21;4:e2807. doi: 10.7717/peerj.2807 (PMC5180584; doi:10.7717/peerj.2807)
Supplement: Table S4 — The results for permutational pairwise tests of levels of the factor Depth are also provided (*: significant outcome after FDR correction). The three layers of each sample pooled. [file peerj-04-2807-s012.docx]

|  | *df* | *SS* | *Pseudo-F* | *P-value* | *Permdisp* |
| --- | --- | --- | --- | --- | --- |
| Season | 1 | 3,916 | 1.662 | 0.009 | 0.022 |
| Depth | 3 | 10,313 | 1.459 | 0.002 | 0.696 |
| Season*Depth | 3 | 7,468 | 1.056 | 0.294 |  |
| Residual | 16 | 37,701 |  |  |  |
|  |  |  |  |  |  |
| **Depth** |  |  |  |  |  |
| *Comparison* | *t* | *P-value* |  |  |  |
| 900 - 1200 | 1.028 | 0.486 |  |  |  |
| 900 - 1500 | 1.271 | 0.019* |  |  |  |
| 900 - 1750 | 1.367 | 0.002* |  |  |  |
| 1200 - 1500 | 1.135 | 0.088 |  |  |  |
| 1200 - 1750 | 1.270 | 0.018* |  |  |  |
| 1500 - 1750 | 1.151 | 0.056 |  |  |  |

Table S4. PERMANOVA and PERMDISP tests of the effects of Season and Depth on the samples from the Blanes Canyon for the Jaccard index. The results for permutational pairwise tests of levels of the factor Depth are also provided (*: significant outcome after FDR correction). The three layers of each sample pooled.
