# Supplementary material for: Spatio-temporal monitoring of deep-sea communities using metabarcoding of sediment DNA and RNA
Source: PeerJ. 2016 Dec 21;4:e2807. doi: 10.7717/peerj.2807 (PMC5180584; doi:10.7717/peerj.2807)
Supplement: Table S5 — The three layers of each sample pooled. [file peerj-04-2807-s013.docx]

|  | *df* | *SS* | *Pseudo-F* | *P-value* | *Permdisp* |
| --- | --- | --- | --- | --- | --- |
| RNA/DNA | 1 | 7,968 | 3.206 | <0.001 | 0.137 |
| Zone | 1 | 6,367 | 2.562 | <0.001 | 0.410 |
| RNA/DNA*Zone | 1 | 2,258 | 0.909 | 0.678 |  |
| Residual | 20 | 49,708 |  |  |  |

Table S5. PERMANOVA and PERMDISP tests of the effects of the type of nucleic acid (RNA or DNA) and Zone (canyon, slope) for the Jaccard index. The three layers of each sample pooled.
